# Fundamental materials engineering of robust solid oxide electrolysis cells for terrestrial and space applications

**DOI:** 10.1039/d6fd00119j

**Published:** 2026-11-12

**Authors:** Robert Price, Alice Sabey, John T. S. Irvine

**Affiliations:** a School of Chemistry, University of St Andrews St Andrews Fife KY16 9ST UK rp63@st-andrews.ac.uk

## Abstract

In this work, a brief summary of the benefits of integrating Rh/Ce_1−*x*_Gd_*x*_O_2−*δ*_ (CGO)-impregnated LSCT_A−_ fuel electrode active layers into commercial standard solid oxide electrolysis cells (SOECs) is provided, before presenting results pertaining to the optimisation of air electrode layers and high-conductivity perovskite electrode current collection layers, which better match the performance and stability of this novel fuel electrode. Firstly, results from solid-oxide electrochemical oxygen pump testing confirmed that ‘traditional’ (La_0.80_Sr_0.20_)_0.95_MnO_3_ (LSM)- and La_0.60_Sr_0.40_CoO_3_ (LSC)-based air electrodes exhibit rapid degradation during oxygen evolution. Replacement with Pr_6_O_11_-impregnated CGO layers, employing LSM and La_0.95_Ni_0.60_Fe_0.40_O_3_ (LNF) as current collectors, significantly improved the durability and oxygen pumping capability of these membranes (for use in a wide variety of applications on Earth and in space). Secondly, alternative fuel electrode current collectors (to Ni(O) layers) were investigated through synthesis, characterisation and electrical testing of a series of La_0.90_Mn_0.95_X_0.05_O_3_ compositions (where X = Ni, Fe, Cu or Ti). The La_0.90_Mn_0.95_Ti_0.05_O_3_ (LMT_A−_) composition showed the highest stability of ‘effective’ electrical conductivity in N_2_ (p(O_2_) ∼10^−5^ atm) and 5% H_2_/95% N_2_ (p(O_2_) = 10^−18^–10^−19^ atm) and was deemed suitable for testing as a fuel-side current collector in SOEC mode. Finally, durability test data for SOECs utilising these optimised air and fuel-side material sets, using a pure carbon dioxide fuel gas, will highlight the system-scale benefits of addressing stability through engineering of electrode materials at nanoscales, for energy conversion technology applications on Earth and in space.

## Abstract 2

Developing robust solid oxide cell electrode materials and microstructures for durable, high-temperature electrochemistry in terrestrial and extra-terrestrial environments.

## Graphical abstract

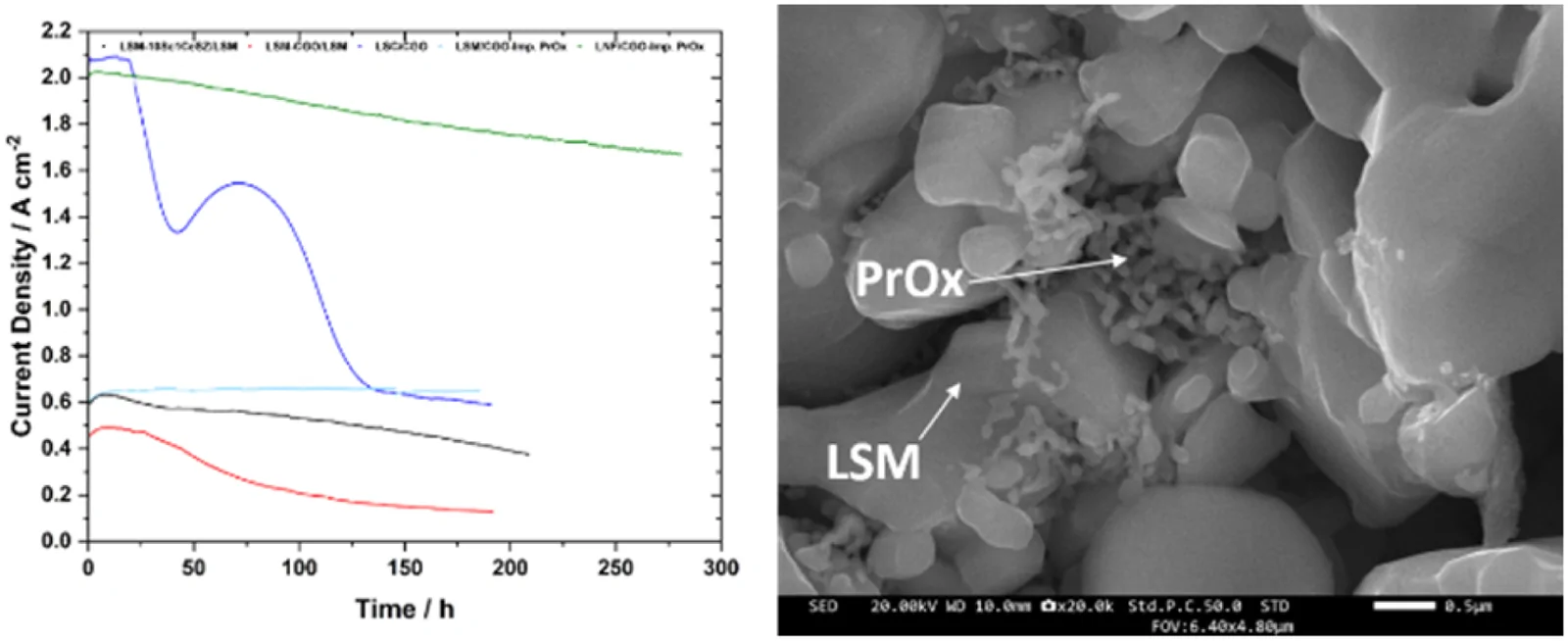

## Introduction

As global concerns regarding climate change, rising energy demand, and ambitious net-zero targets intensify, the development of sustainable technologies for fuel production and energy storage has become increasingly important. Industrial production of CO and H_2_ remains heavily reliant on fossil-fuels, through processes such as steam methane reforming and coal gasification, which cause significant greenhouse gas emissions.^1^ Consequently, there is an urgent need for scalable and durable electrolysis technologies capable of sustainably producing these fuels using renewable energy sources and enabling the decarbonisation of the energy and industrial sectors.

Alongside terrestrial energy challenges, long-term space exploration presents additional demands regarding reliable supplies of oxygen, water, fuel and power, capable of sustaining life in remote environments.^2^ Possible resources available during space exploration include solar power, water and CO_2_ generated by crew respiration, atmospheric CO_2_ and surface or subsurface water-ice.^2,3^ The Mars atmosphere contains high levels of CO_2_, alongside water that can be accessed through the extraction, processing, and melting of surface and subsurface water-ice.^2,3^ These resources can be utilised by electrochemical devices to produce the required oxygen, water, fuel and electrical power for long-term space exploration. Therefore, electrochemical technologies able to operate efficiently and reliably under harsh conditions are of considerable interest for both terrestrial decarbonisation and extra-terrestrial *in situ* resource utilisation (ISRU) applications.

Solid oxide cells (SOCs) are highly efficient, high-temperature (600–900 °C) electrochemical conversion devices comprising two porous electrodes separated by a dense ceramic electrolyte.^4,5^ These systems can operate as solid oxide fuel cells (SOFCs), to generate electrical power and heat from fuel gases, or as solid oxide electrolysis cells (SOECs), to convert steam and carbon dioxide into fuel gases (and oxygen) using electrical energy from renewable resources.^6,7^ Such fuel gases are highly valuable feedstocks in many industrial processes, such as the Fischer–Tropsch process and methanol synthesis.^8^

There are already several examples demonstrating the use of electrolysis technologies in extra-terrestrial applications. Current life-support systems on the International Space Station (ISS) use a proton exchange membrane (PEM)-based electrolyser to produce oxygen from water.^2,10^ Additionally, the use of SOEC technology on Mars was demonstrated by the Mars Oxygen *In Situ* Experiment (MOXIE).^11^ MOXIE successfully converted atmospheric CO_2_ to CO and O_2_ using a SOEC, representing approximately 1% of a full-scale oxygen production system for a human mission.^12^ These successful examples highlight the strong potential of electrolysis technologies for use in long-term space applications.

However, SOECs remain in the early stages of commercialisation^5,7^ and must demonstrate high performance, long operational lifetimes and cost-effective manufacturing in order to achieve widespread deployment.^5,7^ Increasing the lifetime and robustness of these systems remains challenging, particularly in space where maintenance opportunities are extremely limited, and systems must tolerate severe thermal gradients, load cycling and harsh atmospheric gas compositions.

One of the greatest barriers to the widespread commercialisation of SOECs is the degradation of the cell components during long-term operation at high temperatures and under harsh operating conditions.^13,14^ In SOECs, the air electrode is often responsible for some of the most significant degradation pathways. For example, composites of lanthanum strontium manganites (LSM) and stabilised zirconias, which are widely used due to their high electrical conductivity and excellent catalytic activity at temperatures above 800 °C,^15,16^ often exhibit delamination and separation of the ionic and electronic conducting phases due to oxygen bubble formation during operation.^17^ Employment of alternative mixed ionic and electronic conductors, such as lanthanum strontium cobaltite (LSC) and lanthanum strontium cobaltite ferrite (LSCF),^5,7,18^ can circumvent this phenomenon; however, strontium easily segregates from these materials during sintering and high-temperature operation, diffusing through cerium gadolinium oxide (CGO) barrier layers and forming an insulating layer of strontium zirconate at the electrode/electrolyte interface, leading to a decrease in cell performance.^9,19^ At the fuel electrode side, Ni-based cermets such as Ni/YSZ and Ni/Ce_1−*x*_Gd_*x*_O_2−*δ*_ (CGO) are the industrial standards due to their high electrical conductivity and electrochemical activity for fuel gas conversion.^5,7,15^ However, nickel is not stable to redox cycling, leading to volume changes, cracking, and a loss of mechanical integrity,^20–22^ in addition to being highly susceptible to sulfur, carbon and silica poisoning and Ni relocation during high-steam, high-current density operation.^23–26^ Beyond performance issues, nickel mining and processing has negative environmental impacts, including greenhouse gas emissions, water and air pollution, habitat destruction and soil contamination.^27^

In recent years, significant progress has been made in developing non-nickel-containing materials to overcome these challenges. Perovskites are a class of compounds of considerable interest for SOCs as many of them exhibit mixed ionic and electrical conductivity and highly tuneable properties,^28–30^ as well as a plurality of advantages over Ni-based electrodes, such as higher stability and catalytic activity, and increased resistance to carbon deposition with hydrocarbon fuels.^25^ Therefore, these materials are highly promising as alternative fuel electrodes.

Recent work by Price *et al.* on electrolyte-supported SOFCs and SOECs has demonstrated that conventional Ni-based fuel electrodes can be replaced with titanium-based perovskite materials, such as La_0.20_Sr_0.25_Ca_0.45_TiO_3_ (LSCT_A−_), activated through catalyst impregnation with Ce_0.80_Gd_0.20_O_1.90_ and low loadings of Rh nanocatalysts.^24,31^ The titanates have attracted considerable attention as alternative fuel electrodes due to their high stability under a range of atmospheres and their mixed ionic and electrical conductivity.^28,29,32^ In SOFC mode, the LSCT_A−_-based electrodes exhibit full redox and thermoredox stability, the ability to operate in sulfurised natural gas and tolerance to high fuel utilisation or overload conditions at commercial, 1.5 kW system scales.^33^ However, integration of these electrodes into SOECs exposed shortcomings of other cell components including the rapid oxidation of fuel-side Ni(O)-based current collector layers in H_2_O/CO_2_ feeds and air electrodes which degrade during oxygen evolution conditions at high overpotentials.^34^ Consequently, further optimisation of current collection and air electrode materials is required to improve the long-term stability and performance of these LSCT_A−_-based SOEC systems.

In this research, next-generation material sets were evaluated for suitability in solid oxide cell technologies that can be applied in multiple different applications across terrestrial and extra-terrestrial locations. Firstly, Solid-Oxide Electrochemical Oxygen Pumps (SEOPs) were constructed using of ‘state-of-the-art’ (SoA) air electrodes, *e.g.*, LSM and LSC, as well as novel ‘backbone’ electrode microstructures impregnated with Pr_6_O_11_ electrocatalysts. Evaluation of their stability under a harsh operating voltage of 0.4 V was used to determine the stability of these versatile devices, which can be used for separating O_2_ from gas streams for medical applications (oxygen therapy),^35^ generation of propellant oxygen for refuelling of spacecraft^36^ and providing high-purity streams of N_2_ which can be used for ammonia synthesis in the Haber–Bosch process.^37^ In addition, potential alternative fuel electrode current collectors, based upon lanthanum manganites, were investigated due to reports that they exhibit desirable properties including low volume changes during redox cycling, high electrical conductivity, stability in wide p(O_2_) ranges, relatively low cost and being Sr-free. According to work by Ouweltjes *et al.*, La_0.9_Mn_1−*n*_X_*n*_O_3_ (X = Ni, Fe, 0 < *n* ≤ 0.2) compositions with varying levels of Ni and Fe were found to be highly conductive under a reducing 10–90 vol% CO–CO_2_ gas.^22^ Therefore, the current research builds upon this literature report through the evaluation of La_0.90_Mn_0.95_X_0.05_O_3_ (where X = Ni, Fe, Cu or Ti) under lower p(O_2_) conditions. The developments in both air electrode and fuel electrode current collector chemistries were then implemented in full SOECs to determine the effect upon pure CO_2_ electrolysis for the production of fuel (CO) and propellant (O_2_) that could be used to support space exploration in Martian localities.

## Experimental

### Synthesis, characterisation and ceramic processing of La_0.90_Mn_0.95_X_0.05_O_3_ materials

Samples in the La_0.90_Mn_0.95_X_0.05_O_3_ materials series (X = Ni, Fe, Ti, Cu) were prepared *via* a combustion synthesis method. Stoichiometric amounts of La(NO_3_)_3_·6H_2_O (Thermo Scientific, REO, 99.9%), Mn(CH_3_COO)_2_·4H_2_O (Thermo Scientific, 99+ %) and either Ni(NO_3_)_2_·6H_2_O (Thermo Scientific, 99%), Fe(NO_3_)_3_·9H_2_O (Thermo Scientific, ACS Reagent, 98+ %), Cu(NO_3_)_2_·3H_2_O (Sigma-Aldrich, Puriss p.a. 99–104%) or [CH_3_CH(O)CO_2_NH_4_]_2_Ti(OH)_2_ (Sigma-Aldrich, 50 wt% in H_2_O) were dissolved in deionised water and added to a stirred solution of citric acid and ethylene glycol. The pH was reduced to 1 with the addition of nitric acid, and the solution was heated to 150 °C to ensure evaporation of water and formation of a gel. The hotplate temperature was increased to ∼300 °C in order to allow combustion of the ethylene glycol fuel. The resultant black powder was homogenised in a mortar and pestle, placed into an alumina crucible and fired in a muffle furnace at 1100 °C for 2 h.

Samples were analysed by X-ray diffraction (XRD) in the range 2*θ* = 10° to 90° using a Panalytical Empyrean diffractometer, with monochromatic Cu K_α1_ radiation. Diffraction peaks were indexed using STOE WinXPow software and refined using the least squares method to obtain unit cell parameters. The calcined powder was hand ground in a mortar and pestle before undergoing milling for 2 h at 700 rpm in a Fritzsch Pulverisette 7 Planetary Ball Mill with 10 mm diameter zirconia milling media, using ethanol as a solvent. Subsequently, the 10 mm media were removed and replaced by 2 mm diameter media for a final hour of milling to aid particle size reduction and deagglomeration. Particle size analysis (PSA) measurements were carried out using a Malvern Mastersizer 2000 or an Anton Paar 1190L Particle Size Analyser and data analysis was carried out using Mastersizer 2000 (ver. 5.60) software or Anton Paar Kalliope Professional (ver. 3.4.4) software, respectively.

Scanning electron (SE) micrographs were captured in backscattered electron (BSE) mode using a JEOL IT800 Field-Emission Gun Scanning Electron Microscope (SEM) equipped with a JEOL DrySD 30 mm^2^ energy-dispersive X-ray spectroscopy (EDX) detector. Cross-sections of the half-cells and SOCs were vacuum encapsulated in epoxy resin and diamond polished to a 1 µm finish upon curing. The cross sections were coated with silver paint and grounded with Cu tape. Both the cross-sections and powder samples were carbon coated using a Quorum Q150T E Plus Sputter Coater and ultra-high-purity carbon rods.

Screen printing inks were prepared to 70 weight % (wt%) solids loading by combining the ceramic powder with a terpineol (Thermo Scientific, mixed isomers, 98%) solvent and a Hypermer KD1 (Croda) dispersant before planetary ball milling for 2 h at 200 rpm. Subsequently, a polyvinyl butyral (Thermo Scientific, Butvar® B-98) binder was added and the sample was milled for a further 30 min at 150 rpm. Rheological analysis of the screen-printing inks was performed using an Anton Paar MCR72 Rheometer at 20 °C. Shear stress was measured as a function of shear rate over the range 1–200 s^−1^, followed by a reverse sweep from 200–1 s^−1^. An equilibration time of 20 s was applied at each measurement point. The resulting data underwent a Herschel–Bulkley regression to calculate the flow index for samples. Data analysis was carried out using Anton Paar RheoCompass (ver. 1.30) software.

### Preparation of half-cells for four-point conductivity testing

A layer of La_0.90_Mn_0.95_X_0.05_O_3_ ink was screen printed onto a 160 µm thick 6 mol% scandia-stabilised zirconia (6ScSZ, HEXIS AG) electrolyte using a DEK248 semi-automatic screen printer equipped with a 325-mesh screen with a 1 cm^2^ square print geometry. The green layer of ink was applied with a print gap of 1.2 mm and a pressure of 6.0 kg, before drying at 80 °C for 15 min. A second layer of ink was then printed and dried. The samples were sintered up to 1100 °C for 2 h in air within a muffle furnace. Half-cells were prepared by attaching gold wires to the electrode using gold paste. The half-cells were dried at 80 °C for 30 min and then fired in air within a muffle furnace up to 750 °C for 30 min.

The “effective” electrical conductivity of the screen printed samples was measured using a four-point DC conductivity testing rig and custom LabVIEW software. A current was applied between the two outer electrodes, and the resultant voltage was measured between the two inner electrodes. The applied current and measured voltage were used to calculate the resistance, and so the “effective” electrical conductivity could be determined using eqn (1),^38^ where *σ*_eff_ is the “effective” electrical conductivity, *d* is the distance between the electrodes, *R* is the resistance, *l* is the average length of the electrode contacts, and *t* is the thickness of the screen printed layer.1
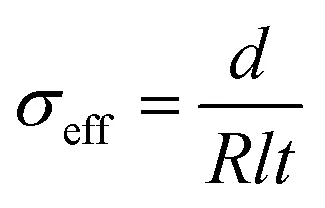


Samples were placed into a tube furnace and heated under a flow of 100 mL min^−1^ compressed air to 850 °C at a ramp rate of 3 °C min^−1^. After ∼24 h, the atmosphere was purged with N_2_ for 1 hour before switching to 5% H_2_/95% N_2_, both supplied at 100 mL min^−1^. Measurements were taken at 60 second intervals using Keithley Model 2401 and 2400 voltage/current sources. The electrode thicknesses were measured using ImageJ analysis software on SEM images of half-cell cross-sections.

### Solid oxide cell preparation

Additional screen printing inks for SEOP and SOEC preparation were either produced directly by industrial SOFC-manufacturer: HEXIS AG or were manufactured at the fuel cell facility pilot production line (Eden Campus, University of St Andrews) using a commercial organic vehicle (terpineol and ethyl cellulose-based) supplied by HEXIS AG, though further details of ink formulation are confidential. Those produced by HEXIS AG included: LSM, composite LSM-10Sc1CeSZ (10 molar% scandia 1 molar% ceria-stabilised zirconia), LSCT_A−_ (Praxair Specialty Ceramics) inks and Ni current collection paste. Those produced in the Fuel Cell Facility included: La_0.60_Sr_0.40_CoO_3_ (LSC), La_0.95_Ni_0.60_Fe_0.40_O_3_ (LNF) – both Praxair Specialty Ceramics – Ce_0.90_Gd_0.10_O_1.95_ (CGO, HEXIS AG), and composite LSM (Praxair Specialty Ceramics) – CGO inks.

SEOPs were manufactured according to the details in Table 1a using an ASMPT DEK NeoHorizon 03iX automatic screen printer to deposit a single layer of air electrode A onto 100 µm thick 6ScSZ (HEXIS AG) electrolytes, before drying up to 200 °C in a BTU Pyramax 75 A convection reflow oven. Two layers of air electrode B were subsequently deposited on top of layer A and dried according to the same procedure. Sintering was performed in accordance with details shown in Table 1a. The same procedure was employed for manufacturing SOECs, though a 140 µm thick 5 molar% scandia-stabilised zirconia (5ScSZ, HEXIS AG) electrolyte was used and the chemistries employed for each SOEC are summarised in Table 1b. Air electrodes were produced in the manner outlined in Table 1a, whilst two layers of LSCT_A−_ ink were deposited onto the opposite side of the electrolyte, before being dried up to 200 °C using a reflow oven. These layers were sintered up to 1100 °C for 2 h, yielding cells with a 1 cm^2^ (square) active area.

A summary of (a) cell chemistries and sintering conditions employed to manufacture SEOPs and (b) the differences in components employed between SOECs tested in this research

**Table:** 

(a) SEOP	Air electrode layer A	Air electrode layer B	Sintering conditions
LSM-10Sc1CeSZ/LSM	LSM-10Sc1CeSZ	LSM	Co-sintered at 1100 °C/2 h
LSM-CGO/LSM	LSM-CGO	LSM	Co-sintered at 1100 °C/2 h
LSC/CGO	CGO	LSC	CGO = 1200 °C/2 h, LSC = unsintered
LSM/CGO – Imp. PrO_*x*_	CGO	LSM	Co-sintered at 1100 °C/2 h
LNF/CGO – Imp. PrO_*x*_	CGO	LNF	Co-sintered at 1100 °C/2 h

**Table:** 

(b) SOEC	Air electrode	Fuel electrode	Fuel electrode current collector
1	LSC/CGO	LSCT_A−_ + CGO + Rh	Ni
2	LSC/CGO	LSCT_A−_ + CGO + Rh	La_0.90_Mn_0.95_Ti_0.05_O_3_
3	LNF/CGO – Imp. PrO_*x*_	LSCT_A−_ + CGO + Rh	La_0.90_Mn_0.95_Ti_0.05_O_3_

The LSCT_A−_-based fuel electrodes were then impregnated with a 0.5 mol per dm^3^ ethanolic solution of Ce(NO_3_)_3_·6H_2_O (Thermo Scientific, 99.5%) and Gd(NO_3_)_3_·6H_2_O (Acros Organics, 99.9%) (dissolved in the required molar ratios to achieve the stoichiometry of Ce_0.80_Gd_0.20_O_1.90_), followed by drying at 80 °C using the reflow oven and calcination up to 500 °C for 30 min. The same procedure was then used to introduce the Rh catalyst to the LSCT/CGO-containing electrode microstructure using a solution of Rh(NO_3_)_3_ (Johnson-Matthey) diluted with ethanol. The process was also used to add Pr_6_O_11_ catalyst particles into air electrodes with LNF/CGO or LSM/CGO microstructures (SEOPs and SOEC 3) using a 0.5 mol per dm^3^ ethanolic solution of Pr(NO_3_)_3_·5H_2_O (Thermo Scientific, 99.9%). Finally, if required, two layers of air electrode (LSC) and fuel electrode (Ni or La_0.90_Mn_0.95_Ti_0.05_O_3_) current collection inks were deposited onto the active fuel electrode layers using screen printing, before drying up to 200 °C using the reflow oven, and were left unsintered. Schematics for the construction of SEOPs and each SOEC are provided in Fig. 1.

**Fig. 1:**
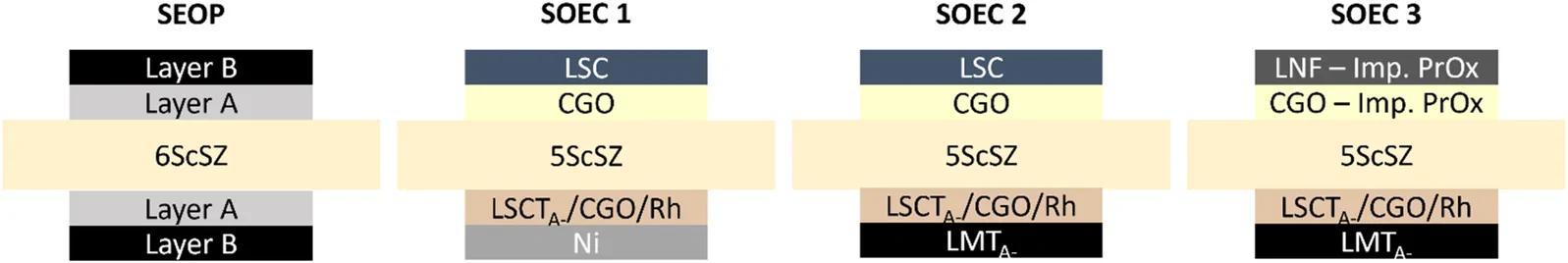
A schematic for the general components used to manufacture the SEOPs, as well as specific components used to manufacture SOECs 1, 2 and 3.

### Solid oxide cell testing

The SOC test setup consisted of an alumina cell housing, divided into upper and lower sections. A gold mesh welded to platinum wires was used to collect current from each electrode. The SOCs were positioned in the lower section of the alumina housing and sealed by alumina felt gas diffusion gaskets placed on both sides of the cells. Finally, the upper section of the housing was positioned on top of the cell. SOCs were heated to the required operating temperature in air at a rate of 1 °C min^−1^. Upon reaching the target temperature, the fuel and air electrode compartments were purged with N_2_ at a 200 mL min^−1^ flow rate. In the case of SEOP testing, a flow of 200 mL min^−1^ of compressed air was then supplied to each electrode, whilst the supply of N_2_ to this compartment was stopped. For SOEC testing, 200 mL min^−1^ flows of air and CO_2_ were supplied to the air and fuel electrodes, respectively, before stopping the N_2_ purges. SEOP testing involved collecting voltage–current (*VI*) curves, between open circuit voltage (OCV) and 0.5 V, as well as AC impedance spectra at 0.5 A cm^−2^ (between 20 KHz and 0.2 Hz with an excitation amplitude of 20 mA) at 850 °C. These measurements were taken before and after potentiostatic durability testing, which was conducted at the same temperature and at 0.4 V. Finally, for SOEC testing, cells were heated to 750 °C before initial electrochemical evaluation of cells was conducted in the form of *VI* curves (OCV to −2.0 V) and AC impedance spectra at −1.5 V between 20 kHz and 0.2 Hz, with an excitation amplitude of 20 mV. This data was collected between 750 °C and 850 °C at 25 °C intervals. Following this initial characterisation, potentiostatic measurements were conducted at −1.47 V for up to 310 h. *VI* curves and AC impedance spectra were collected under the same conditions after the durability testing period. All *VI* curves and AC impedance spectra were collected using a Solartron SI 1280B Electrochemical Measurement Unit, with CorrWare (ver. 3.5I) and Zplot (ver. 3.5I) software, whilst potentiostatic operation (with 300 s between data points) was performed using a Solartron 1286 potentiostat with custom LabVIEW data collection software.

## Results and discussion

### Developing stable electrodes for oxygen separation in solid-oxide electrochemical oxygen pumps

In order to demonstrate the robustness and stability of high-temperature oxygen pumping, for the ultimate purposes of generating pure streams of oxygen for medical/life support applications or spacecraft propellants, or indeed removal of oxygen to create pure streams of N_2_ for ammonia synthesis, Solid-Oxide Electrochemical Oxygen Pumps (SEOPs) were manufactured and tested. The SEOPs consisted of a 6ScSZ electrolyte sandwiched between two air electrodes, comprising either ‘traditional’ state-of-the-art material sets or next-generation air electrodes. Testing was performed for 281 h at 850 °C and a harsh operating voltage of 0.4 V to determine which material sets could offer the required stability for long-term. Here, one air electrode undertakes the oxygen reduction reaction (ORR), like that experienced by a SOFC cathode, whilst the other facilitates the oxygen evolution reaction (OER), like that experienced by a SOEC anode, providing valuable information on the stability of each electrode in different operational modes whilst pumping oxygen across the ceramic membrane. Fig. 2 displays a plot of current density *versus* time for the five SEOPs tested in this work. It is possible to see the composite materials (*e.g.*, LSM-10Sc1CeSZ/LSM and LSM-CGO/LSM) show similar behaviour and after an initial equilibration period of ∼7 h, severe degradation of current density ensues and does not cease. Typically, in this type of composite air electrode, oxygen bubble evolution occurs between grains of the ionic conductor (*e.g.*, 10Sc1CeSZ) and electronic conductor (*e.g.*, LSM) and can force them apart, leading to delamination of the air electrode, particularly when oxygen bubbles evolve in closed pores at the air electrode–electrolyte interface.^17,39–42^ This is a widely reported phenomenon in the literature and also aligns well with the authors' previous work on SOECs, in which degradation was attributed primarily to the LSM-based air electrodes.^31^

**Fig. 2:**
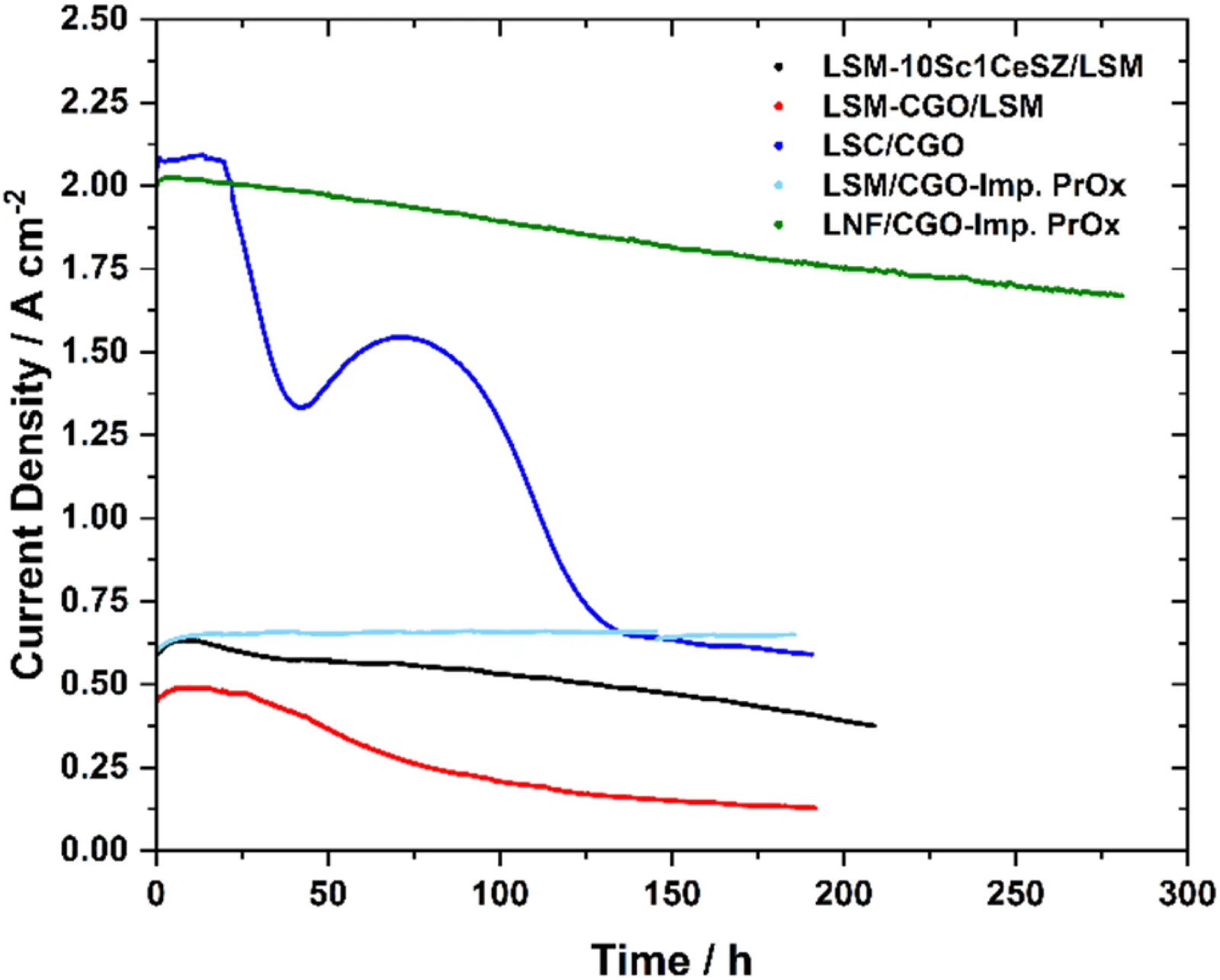
A plot of current density *versus* time for SEOPs under potentiostatic operation at 0.4 V (at 850 °C with compressed air supplied to both electrodes), showing the degradation behaviour of ‘traditional’ material sets in comparison to those modified by catalyst impregnation.

Fig. 3a and b display *VI* curves for each cell collected before and after SEOP testing, respectively, whilst Fig. 4 shows the complex plane AC impedance spectra collected at 0.5 A cm^−2^ before and after SEOP testing for each material set. The *VI* curves confirm that severe degradation in current passage occurred as a result of operation for the LSM-10Sc1CeSZ/LSM and LSM-CGO/LSM SEOPs, but in particular, significant increases in series resistance (*R*_s_) and polarisation resistance (*R*_p_) can be observed in the AC impedance spectra. This may be the result of a decrease of contact at the surface of the electrode, *i.e.*, current collection issues, or a reduction of contact at the electrode–electrolyte interfaces, pointing towards delamination of the oxygen evolution electrode. If the ratio of *R*_s_/*R*_p_ (Fig. 4) remains constant before and after the testing period, a scaling of the impedance response has occurred, which points to delamination or loss of contact as a key cause of the observed degradation, and appears to be the case for most SEOPs in this research. This is a well-documented phenomenon when operating above the critical current density window of 0.5–1.0 A cm^−2^.^34,43^ It is also possible that segregation of SrO to the surface of the LSM phase under anodic polarisation (*i.e.*, at the OER electrode) could occur in these SEOPs, reducing the activity for oxygen evolution.^44^ Upon removal from the test setup, the oxygen evolution electrodes of these SEOPs were either very fragile or had delaminated from the electrolyte, as shown in the SE micrographs of epoxy-cured and polished cross-sections of these SEOPs in Fig. 5a and b.

**Fig. 3:**
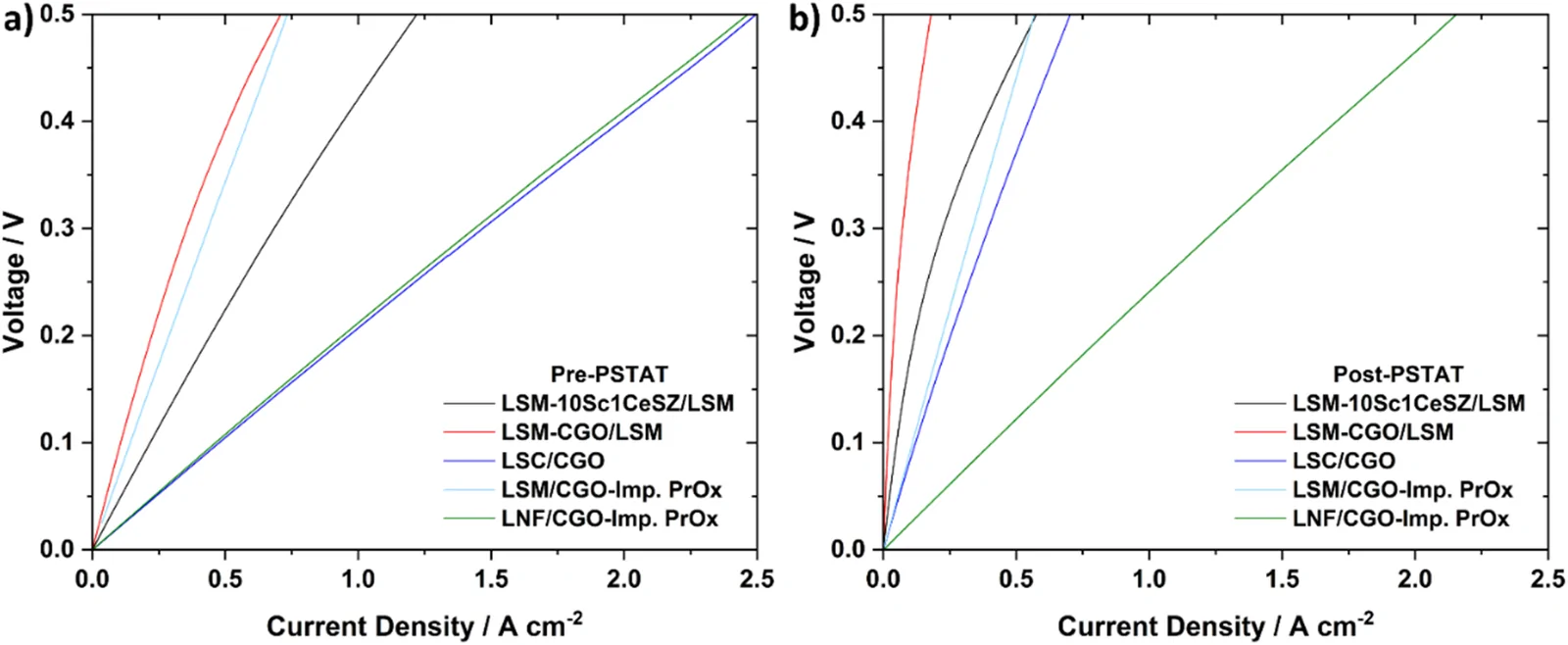
Voltage–current (*VI*) curves for each SEOP collected (a) before and (b) after durability testing.

**Fig. 4:**
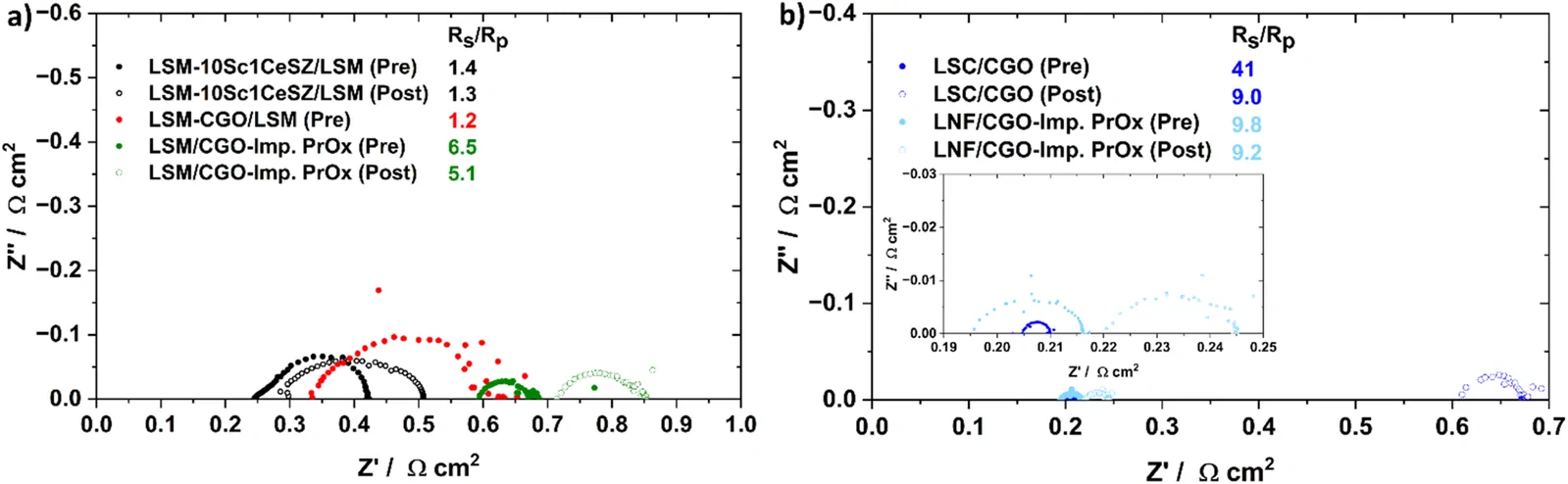
AC impedance spectra collected at 0.5 A cm^−2^ current density taken before and after durability testing for (a) LSM-10Sc1CeSZ/LSM, LSM-CGO/LSM and LSM/CGO – impregnated with Pr_6_O_11_ and (b) LSC/CGO and LNF/CGO – impregnated with Pr_6_O_11_. Please note that a representative post-test AC impedance spectrum could not be collected for the LSM-CGO/LSM SEOP.

**Fig. 5:**
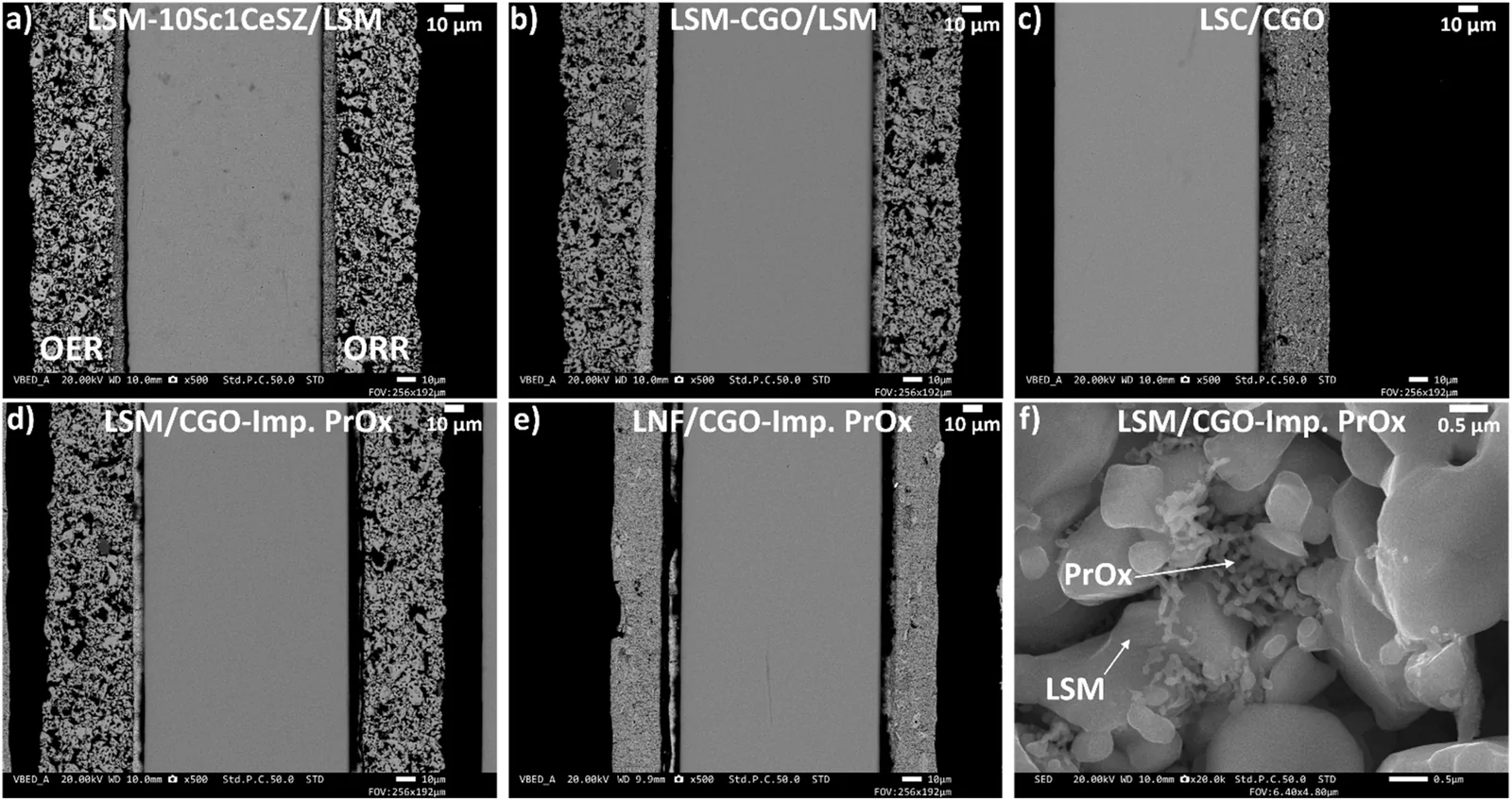
Post-mortem BSE micrographs of polished cross sections of SEOPs with air electrodes of (a) LSM-10Sc1CeSZ/LSM, (b) LSM-CGO/LSM, (c) LSC/CGO, (d) LSM/CGO – impregnated with Pr_6_O_11_ and (e) LNF/CGO – impregnated with Pr_6_O_11_ (OER electrodes face left and ORR electrodes face right, as shown in (a)); (f) displays a high-magnification SE micrograph of a broken cross-section of (d), with the location of LSM grains and Pr_6_O_11_ nanoparticles denoted.

To mitigate this effect, mixed ionic and electronic conducting (MIEC) materials, *e.g.*, LSC or LSCF, were developed^45,46^ to provide higher electrocatalytic activity for oxygen reduction/evolution, lower air electrode overpotentials and ensure that oxygen evolution only occurs at the surface of a single-phase material. However, Sr readily segregates from these materials and diffuses to the air electrode–electrolyte interface, mainly during sintering but also during operation. Despite the application of dense CGO barrier layers between the air electrode and electrolyte, Sr is still able to diffuse to this interface where it can form an electrically insulating layer of SrZrO_3_, which severely limits performance of SOECs.^31^ Prior work has demonstrated that sintering of LSCF layers even at relatively low temperatures of 1060 °C results in the formation of SrZrO_3_ layers in industrially manufactured SOECs, which limits performance despite reducing the severity of degradation.^31^ An alternative option is to leave this MIEC layer unsintered to prevent excessive formation of insulating layers. Fig. 2 displays the behaviour of a SEOP with CGO barrier layers and unsintered LSC MIEC layers. The initial current density of this SEOP is more than three times higher than for the LSM-10Sc1CeSZ/LSM SEOP, due to the increased bulk electrical conductivity (*σ*_e_ = 1585 S cm^−1^ at 800 °C) and ionic conductivity (*σ*_i_ = 0.22 S cm^−1^ at 800 °C)^9^ of LSC in comparison to LSM (*σ*_e_ = 152 S cm^−1^ and *σ*_i_ = 4.0 × 10^−8^ S cm^−1^ at 900 °C).^9^ In similarity to all other samples, there is an initial equilibration period over the course of the first 12 h of operation, during which the current density gradually rises. However, catastrophic degradation begins at ∼20 h which reduces the current density from 2.09 A cm^−2^ at 14 h to 0.59 A cm^−2^ after 191 h. Between 43 and 71 h, there was an unexpected rise in current density, succeeded by a rapid fall, which may relate to the low stability of the LSC material under air starvation conditions. This has been observed in SOFCs at stack/system scales when air supply failures occur and current continues to be drawn from the stack.^47^ Here, the p(O_2_) at the air electrode–electrolyte interface can drop as low as 10^−6^ atm,^47^ where LSC can rapidly decompose. This phenomenon may be occurring in the current work due to oxygen depletion at the ORR electrode as a result of high-current-density operation and harsh operating voltage.

The *VI* curves (Fig. 3) collected before and after durability testing of the LSC-CGO SEOP show the greatest reduction in current passage of all cells tested, which is also reflected by the corresponding AC impedance spectra. For this SEOP, there is large difference in *R*_s_/*R*_p_ ratios before and after the testing period. Therefore, although delamination is most likely the key cause of degradation in this SEOP, changes in electrode performance as a result of the low stability of LSC under slightly reducing conditions may have contributed to degradation. Post-mortem microscopic examination of this SEOP (Fig. 5c) confirmed that the OER electrode had also completely delaminated from the electrolyte, which is not surprising given the combination of initial high-current-density operation (*i.e.*, high oxygen evolution) and a relatively weakly adhering LSC layer due to the lack of a high-temperature sintering step. Therefore, when utilising LSCF or LSC-based air electrodes, a compromise must be struck between achieving good MIEC layer adhesion, with high-temperature sintering, and SrZrO_3_ formation at the air electrode–electrolyte interface.

Alternatively, next-generation material sets must also be trialled to determine whether the limitations of traditional material sets can be overcome. Given the success of implementation of catalyst impregnation to create highly intricate, electrochemically active and stable nanostructures in Rh/CG20 impregnated LSCT_A−_ fuel electrodes, the same approach has been applied to air electrodes for many years.^37,48–55^ In this research, additional SEOPs comprising a porous CGO layer, with either a LSM or LNF current collection layer, were manufactured and sintered. Subsequently, both air electrodes were impregnated with Pr(NO_3_)_3_·5H_2_O solution to introduce Pr_6_O_11_ into the electrode microstructures, after calcination. The SEOP containing LSM/CGO electrodes, impregnated with 985 µg cm^−2^ Pr_6_O_11_ per electrode, shows a similar initial current density to the LSM-10Sc1CeSZ/LSM SEOP; however, performance stabilises over the course of the 186 hour test period. This most likely arises as the sites of oxygen evolution reaction have moved from the triple phase boundary (TPB) between the LSM, 10Sc1CeSZ and gas phase to a double phase boundary (DPB) between CGO and/or Pr_6_O_11_ and the gas phase, circumventing issues with delamination. A SE micrograph of the broken cross-section this particular oxygen evolution electrode shows that the impregnated Pr_6_O_11_ catalyst particles are approximately 100 nm in diameter and interconnect to other particles within the LSM scaffold area of the electrode (Fig. 5f). Considering the *VI* curves and AC impedance spectra, although the initial performance is not high in comparison to the LSC/CGO SEOP, little degradation is observed over this time period, which is encouraging for improving the stability of the oxygen separation process. The final SEOP contained a LNF current collection layer, rather than LSM, and each electrode was impregnated with 545 µg cm^−2^ Pr_6_O_11_, resulting in a much higher initial performance, akin to that of the traditional LSC/CGO SEOP. However, the degradation has been significantly decreased over a longer (281 hour) period.

Fig. 5 displays SE micrographs of polished cross-sections of each SEOP that underwent durability testing. Delamination of the electrodes can be seen in all cases; however, for SEOPs displayed in Fig. 5a–c, delamination occurred during disassembly from the test rig, whilst for those shown in Fig. 5d and e, delamination occurred during the cutting and polishing process and so was not related to operation. All LSM current collection layers (Fig. 5a, b and d) show good adhesion to the underlying functional/active layers and the microstructures are homogeneous, yet coarse, which is desirable to maximise electrical conduction through the bulk of the larger LSM grains. This is particularly important in maximising the ‘effective’ electrical conductivity of porous LSM microstructures, when this material displays significantly lower values (*e.g.*, 370 S cm^−1^ at 850 °C in air – presented in following sections of this manuscript) than the bulk conductivity of LSC (1585 S cm^−1^ at 800 °C).^9^ The LSC layer exhibits a slightly finer microstructure (Fig. 5c), though larger particles are still present to help it perform its function as a current collection layer. In contrast, the LNF current collection layer (Fig. 5e) exhibits a much finer microstructure.

Despite the fact that LNF exhibits a bulk conductivity of approximately 370 S cm^−1^ at 850 °C in air, according to work by Konysheva *et al.* (similar to LSM above), this SEOP gave rise to the highest initial current density, with improved stability. Interestingly, its conductivity under inert gas atmospheres remains high at approximately 320 S cm^−1^ at 850 °C, making it ideal for use in ORR electrodes in SEOPs, where oxygen depletion may occur.^56^ This particular air electrode is, therefore, advantageous to use in SEOPs, which require high robustness, and where maintenance opportunities are limited (*e.g.*, in space exploration applications) and may also be deployed as a stable air electrode for pure CO_2_ electrolysis, which shares a similar issue with degradation.

### Investigation of transition-metal-doped La_0.90_Mn_1−*n*_X_*n*_O_3_ materials as fuel electrode current collectors

#### Synthesis, physical characterisation and ceramic processing of La_0.90_Mn_0.95_X_0.05_O_3_

A variety of lanthanum manganite-based materials containing B-site dopants of Ni, Fe, Cu and Ti, based upon the formula La_0.90_Mn_0.95_X_0.05_O_3_, were synthesised using a combustion synthesis method, before being calcined up to 1100 °C in air. The compositions of each material and abbreviations are summarised in Table 2a. All samples were analysed by pXRD and the resultant patterns are displayed in Fig. 6. Fig. 6a displays the pXRD patterns of the full range analysed (10–90° 2*θ*), whilst Fig. 6b shows a magnified region, which clearly demonstrates a shift in peak position of the reflection at ∼32.5° as a function of B-site dopant. Lattice parameters were determined through unit cell refinement and, at room temperature, all samples were phase pure with rhombohedral symmetry, pertaining to the *R*3̄*c* space group. This is consistent with literature findings for similar lanthanum manganites;^57,58^ however, the specific work performed by Ouweltjes *et al.* suggests that the LMN_A−_ and LMF_A−_ materials pertain to an orthorhombic space group,^22^ though no further structural characterisation data is provided in this case. The determined lattice parameters for each material are listed in Table 2a, indicating, again, that transition-metal-doping was successful, as shown by the change in *a*, *c* and cell volume parameters as a function of dopant. Following confirmation of the phase purity of the target materials, ceramic processing of these lanthanum manganites was undertaken. Initially, PSA and SEM were carried out in order to provide insights into the particle size and morphology of the raw powders, before high-speed planetary ball milling was employed to reduce their particle sizes and ensure comparable particle size distributions between materials. Fig. 7a shows an example of the particle size reduction process during ball milling for the LMT_A−_ material over the course of 3 h of milling at 700 rpm. As the raw LMT_A−_ powder contained large particles up to 300 µm in diameter, the first hour of ball milling was crucial in breaking down weakly bound agglomerates formed during calcination. Following this step, particle size continued to reduce as a function of milling time with the 10 mm diameter media (second hour) and 2 mm diameter media (third hour). A summary of the *D*_50_ values (the diameter below which 50% of the volume fraction of the sample sits) for each material before and after milling is included in Table 2b.

(a) Refined unit cell parameters for lanthanum manganite-based materials synthesised and (b) *D*_50_ values for raw and milled lanthanum manganite-based powders obtained from particle size analysis

**Table:** 

(a) Material	Abbreviation	*a*, *b*/Å	*c*/Å	Cell volume/Å^3^
LaMnO_3_	LMO	5.5270(3)	13.3379(5)	352.85(3)
La_0.90_MnO_3_	LMO_A−_	5.5268(3)	13.3611(4)	353.44(3)
La_0.90_Mn_0.95_Ni_0.05_O_3_	LMN_A−_	5.52379(16)	13.3421(3)	352.557(18)
La_0.90_Mn_0.95_Fe_0.05_O_3_	LMF_A−_	5.5229(4)	13.3912(6)	353.74(4)
La_0.90_Mn_0.95_Cu_0.05_O_3_	LMC_A−_	5.5267(3)	13.3488(4)	353.11(3)
La_0.90_Mn_0.95_Ti_0.05_O_3_	LMT_A−_	5.5290(3)	13.3718(4)	354.01(3)

**Table:** 

(b) Material	D_50_ raw powder/µm	D_50_ milled powder/µm
LaMnO_3_	33.851	1.681
La_0.90_MnO_3_	33.395	1.786
La_0.90_Mn_0.95_Ni_0.05_O_3_	27.189	1.905
La_0.90_Mn_0.95_Fe_0.05_O_3_	35.639	1.778
La_0.90_Mn_0.95_Cu_0.05_O_3_	36.233	1.731
La_0.90_Mn_0.95_Ti_0.05_O_3_	28.327	1.692

**Fig. 6:**
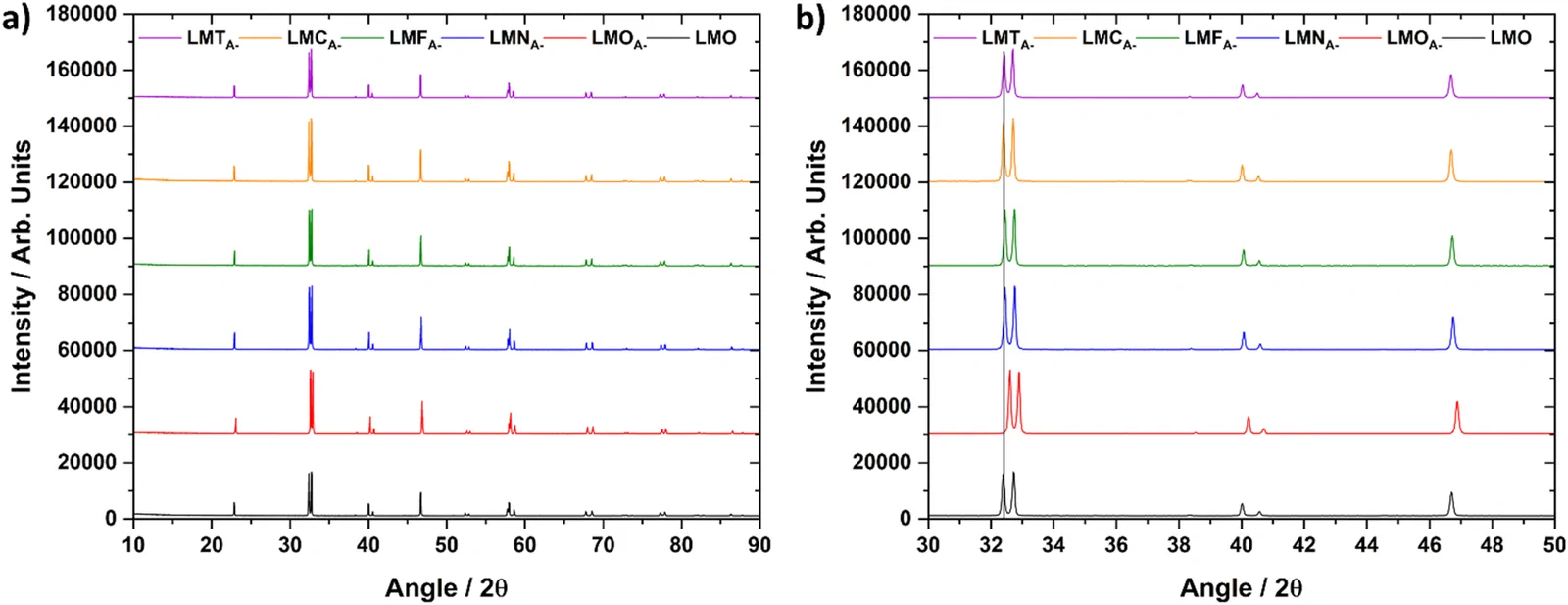
Powder X-ray diffraction patterns for the La_0.90_Mn_0.95_X_0.05_O_3_ series with varying transition metal dopants (a) over the 10–90° 2*θ* range and (b) a magnified region (30–50° 2*θ*) with a vertical line at ∼32.5° to highlight the peak shift as a function of dopant.

**Fig. 7:**
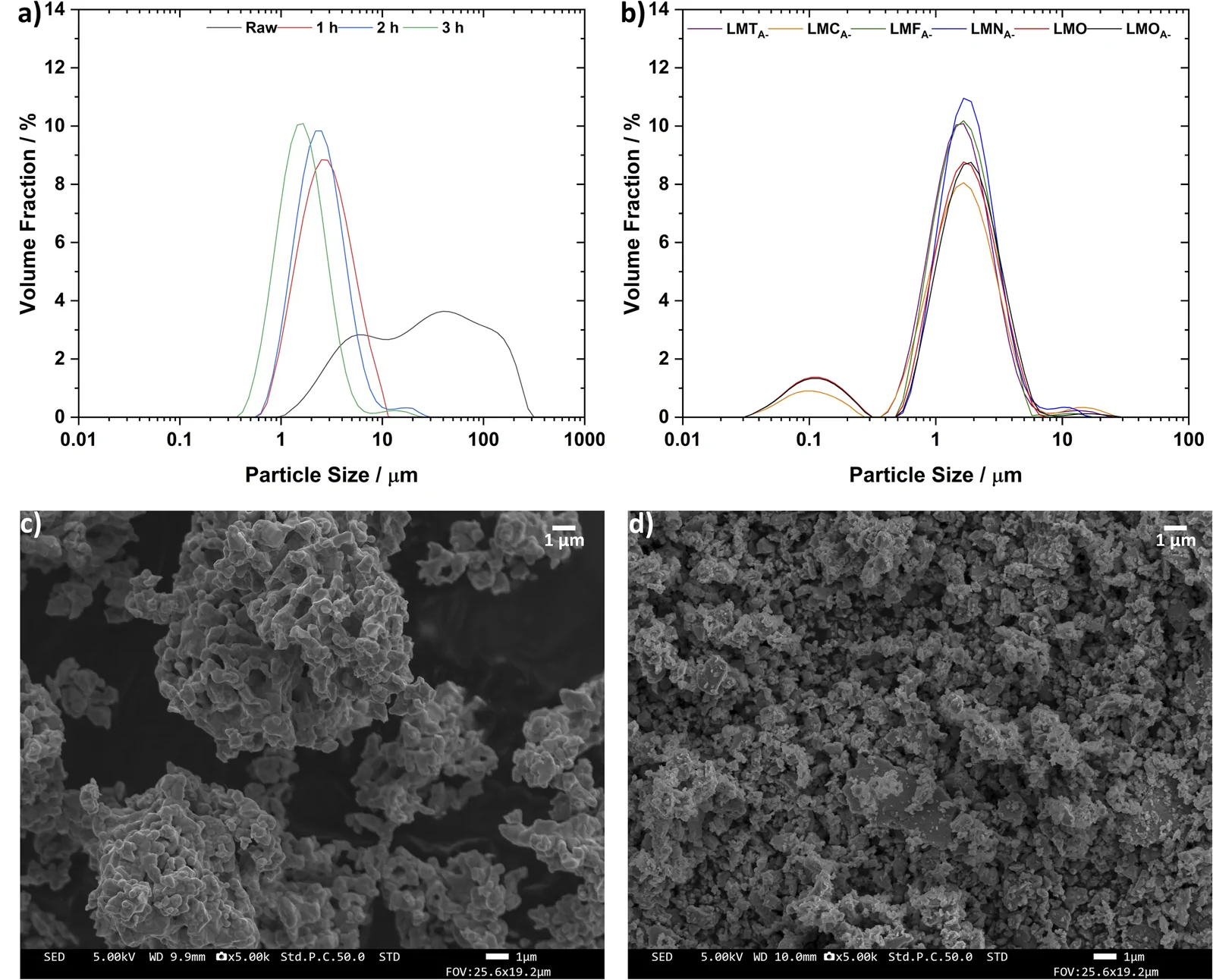
Particle size distributions (a) as a function of high-speed planetary ball milling time for LMT_A−_ and (b) for all lanthanum manganite materials after 3 h of ball milling, and scanning electron micrographs of the (c) the raw LMT_A−_ powder (as synthesised) and (d) the LMT_A−_ powder after 3 h of ball milling.

This is also confirmed by differences in particle size and morphology observed in SE micrographs of the raw *versus* milled LMT_A−_, presented in Fig. 7c and d, respectively. The same progression was observed for LMO, LMO_A−_, LMN_A−_, LMF_A−_, and LMC_A−_ materials (Fig. 7b) and after 3 h of milling, each of the samples exhibited a unimodal distribution (∼1.7 µm), with the exception of LMO, LMO_A−_ and LMC_A−_, which also displayed small finer fractions ∼0.1 µm. The careful optimisation of PSDs of materials for electrochemical devices is an extremely important factor in developing high-quality, well-dispersed inks that can be used to screen print homogeneous, consolidated, thick-film layers for use as electrodes in SOCs. Each component (*e.g.*, electrodes, electrolytes or current collectors) may be produced using different ceramic processing methods (*e.g.*, screen printing, tape casting, spray pyrolysis) and requires different physical characteristics of the ceramics. In the case of manufacturing effective current collection layers, smaller particle sizes help densification, and a narrow particle size distribution improves particle packing density and dispersion of particles in a suspension (*i.e.*, a screen printing ink).^59^

The milled ceramic powders were then incorporated into screen printing inks using a two-stage planetary ball milling process: (i) a dispersion milling step at ∼200 rpm and (ii) a binder mixing step at ∼150 rpm. Inks with 70 wt% solids loading of each lanthanum manganite-based material were made using this method to provide comparability between materials post-screen printing and sintering. The rheological behaviour of the inks was then analysed in order to confirm that the inks possessed desirable characteristics for the screen printing process. Plots of shear stress *versus* shear rate are presented in Fig. 8a along with examples of flow indices determined. This provides insight into the fluid-dynamic behaviour of an ink upon application of shear stress. A flow index equal to 1 corresponds to Newtonian flow, where viscosity remains constant with shear rate. Values greater than unity show dilatant (shear-thickening) behaviour, while values less than unity exhibit pseudoplastic (shear-thinning) behaviour.^60^ The flow indices in Fig. 8a (0.86–0.87), indicate pseudoplastic (shear-thinning) behaviour, which is an ideal property for screen printing.^60,61^ Here, as the movement of the squeegees shears the ink, a reduction in viscosity occurs, allowing the ink to flow through the porous screen geometry and onto the substrate below, leaving a homogeneous and well-defined layer behind.^62^

**Fig. 8:**
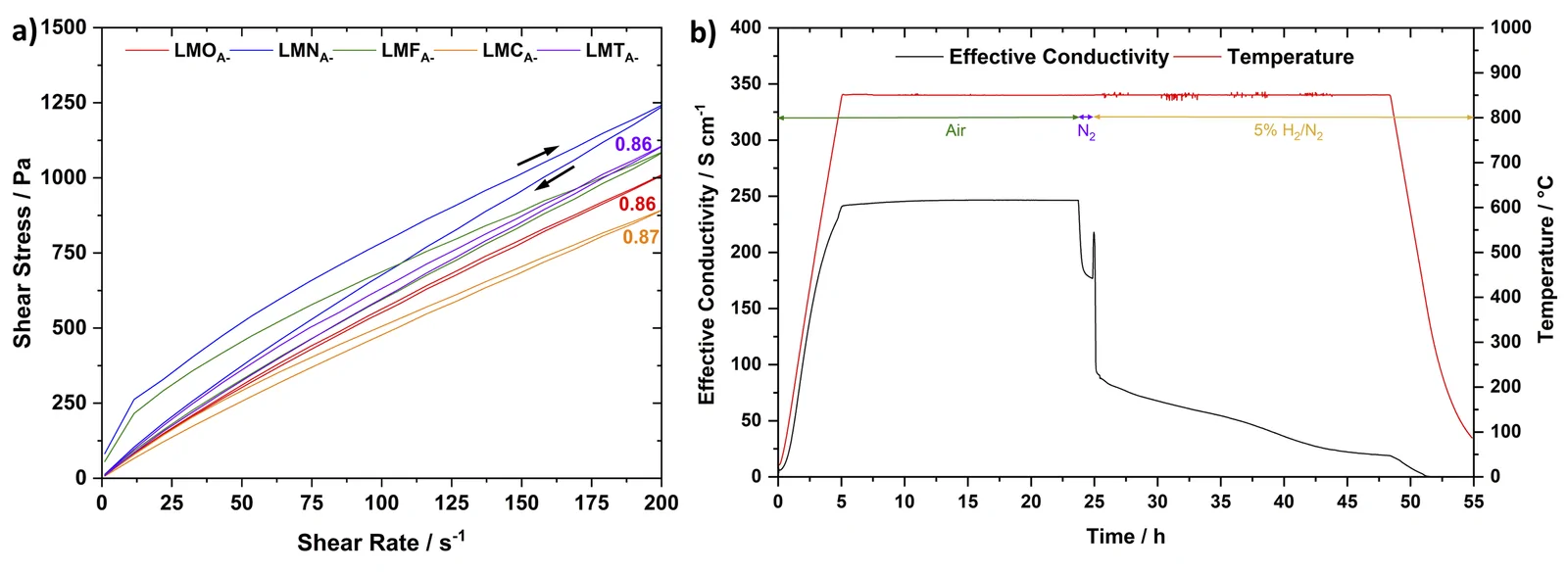
(a) A plot of shear stress *versus* shear rate for a variety of La_0.90_Mn_0.95_X_0.05_O_3_ inks, with accompanying flow indices, and (b) a plot of effective conductivity and temperature *versus* time for the LMF_A−_ half-cell.

In addition the hysteresis between the upscan and downscan data series indicates that some thixotropic (time-dependent shear-thinning) behaviour occurs, which is also useful in allowing levelling of screen printed layers after the screen mesh has snapped away from the substrate.^60^ Half-cells were then manufactured by depositing 2 layers of ink of each lanthanum manganite-based material onto separate 20 mm diameter 6ScSZ electrolytes, of 160 µm thickness, before sintering at 1100 °C for 2 h in ambient air. It is important to note that the sintering of these layers is essential in allowing Au electrodes to be attached to the surface of the thick-films without removal of the underlying layer, though this differs from the way current collection layers are often applied during SOC manufacture.

#### Electrical characterisation of thick-film La_0.90_Mn_0.95_X_0.05_O_3_ layers

The effective electrical conductivity of thick-film layers of each material was measured using this half-cell configuration in compressed air, nitrogen and 5% H_2_/95% N_2_ (forming gas) to identify the most suitable candidates to be trialled in SOC testing. Effective conductivity takes into account the porosity and grain boundaries present in SOC electrodes and provides a more appropriate evaluation of the conductivity behaviour in a real SOC electrode or current collection layer. It is, therefore, not directly comparable to bulk conductivity values for similar materials sets, which have been compressed and sintered to >98% of their theoretical density. Fig. 8b shows a plot of effective conductivity and temperature *versus* time for the LMF_A−_ half-cell, which is a representative example of the experiment undertaken for each material.

Upon heating from ∼50 °C to 850 °C in compressed air (required for SOC operation), the effective conductivity increases, displaying semiconductive behaviour. It is well documented that lanthanum manganite-based materials exhibit p-type conductive behaviour in which ‘holes’ act as the charge carriers.^9^ Once the target temperature was reached, the conductivity stabilised, at 247 S cm^−1^, as expected due to the excellent stability of these material sets at high operating temperatures in air (p(O_2_) ∼0.21 atm). Before switching to forming gas, a N_2_ purge (p(O_2_) ∼10^−5^ atm) was performed for approximately one hour, causing a rapid drop in conductivity. After switching to forming gas (p(O_2_) = 10^−18^–10^−19^ atm), and the clearance of a small volume of air in the system (indicated by a brief increase in p(O_2_)) due to manual switching of gas lines, the conductivity exhibited another significant decrease, reducing to 84 S cm^−1^ only one hour after this introduction. The conductivity did not stabilise throughout the remainder of the test period, prior to cooling down after 48 h of testing.

Fig. 9a presents a comparison of the effective conductivities of the La_0.90_Mn_0.95_X_0.05_O_3_ materials investigated in this study, along with (La_0.80_Sr_0.20_)_0.95_MnO_3_ (LSM) as a reference material, upon heating to 850 °C in compressed air. LMO_A−_ achieved the highest effective conductivity of 446 S cm^−1^ at 850 °C in air, followed by LSM (370 S cm^−1^) and LMC_A−_ (341 S cm^−1^). Though a software issue prevented data collection during heating from 462 °C to 850 °C for the LMC_A−_ sample, this conductivity value was recorded once data collection was restarted during the 850 °C dwell period.

**Fig. 9:**
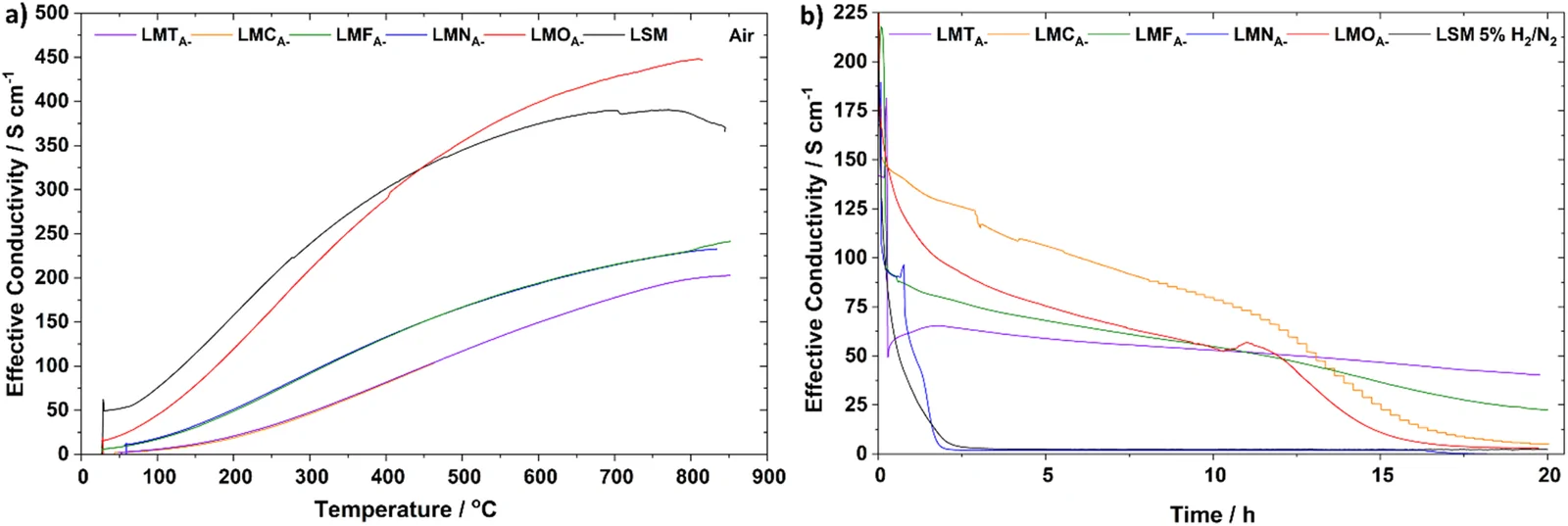
Plots of (a) effective conductivity *versus* temperature for the La_0.90_Mn_0.95_X_0.05_O_3_ thick-films upon heating to 850 °C in compressed air and (b) effective conductivity *versus* time in forming gas, showing the extent of stability of these materials under reducing conditions.

Upon exposure to 5% H_2_/N_2_, the conductivity of all samples showed rapid degradation (Fig. 9b). The conductivity of Ni-cermet-based fuel electrodes under reducing conditions is typically greater than 1000 S cm^−1^;^63^ however, after 20 h exposure to reducing conditions, the effective conductivities of LMO_A−_, LMN_A−_ and LMC_A−_ had reduced to <5 S cm^−1^. While achieving conductivities comparable to nickel may not be feasible for these materials, a current collector for use in a SOC with a LSCT_A−_ fuel electrode should at least exceed the effective conductivity of the electrochemically active electrode material (17 S cm^−1^ at 850 °C).^61^ Therefore, these materials were deemed unsuitable for use as current collectors due to their low electrical conductivity.

Interestingly, both LSM and LMN_A−_ showed similar conductivity decay profiles. Under reducing conditions, similar LSM materials (La_0.6_Sr_0.4_MnO_3_) decompose into a Ruddlesden–Popper phase (La_0.6_Sr_0.4_MnO_4+*δ*_) and MnO_*x*_.^64^ Given the similarity of the lanthanum manganite-based materials sets synthesised in this work, this may provide an explanation as to the primary degradation pathway of conductivity observed in Fig. 9b.

Although the behaviour of LMN_A−_ and LMF_A−_ was very similar under oxidising conditions, the LMF_A−_ sample exhibited significantly better stability over the same period under reducing conditions. However, although the LMT_A−_ material exhibited the lowest effective conductivity in air, under a reducing atmosphere it demonstrated much higher stability than all other samples. After 20 h in 5% H_2_/N_2_, LMT_A−_ retained a value of 40 S cm^−1^ making LMT_A−_ the most promising material to be evaluated as a current collector layer in a SOEC.

Post-mortem SE microscopy was performed on polished cross-sections of half-cells in order to determine the thickness of the layers (for conductivity calculations). Fig. 10 displays BSE micrographs for: (a) LMO_A−_, (b) LMN_A−_, (c) LMF_A−_, (d) LMC_A−_, (e) LMT_A−_ and (f) LSM. There were no major differences in density between the electrodes and good adhesion of the layers to the electrolyte was observed. However, even from the low magnification micrograph in Fig. 10f, regions of segregation within the microstructure can be seen, which may relate to the aforementioned Ruddlesden–Popper and MnO_*x*_ decomposition products of LSM under reducing conditions.

**Fig. 10:**
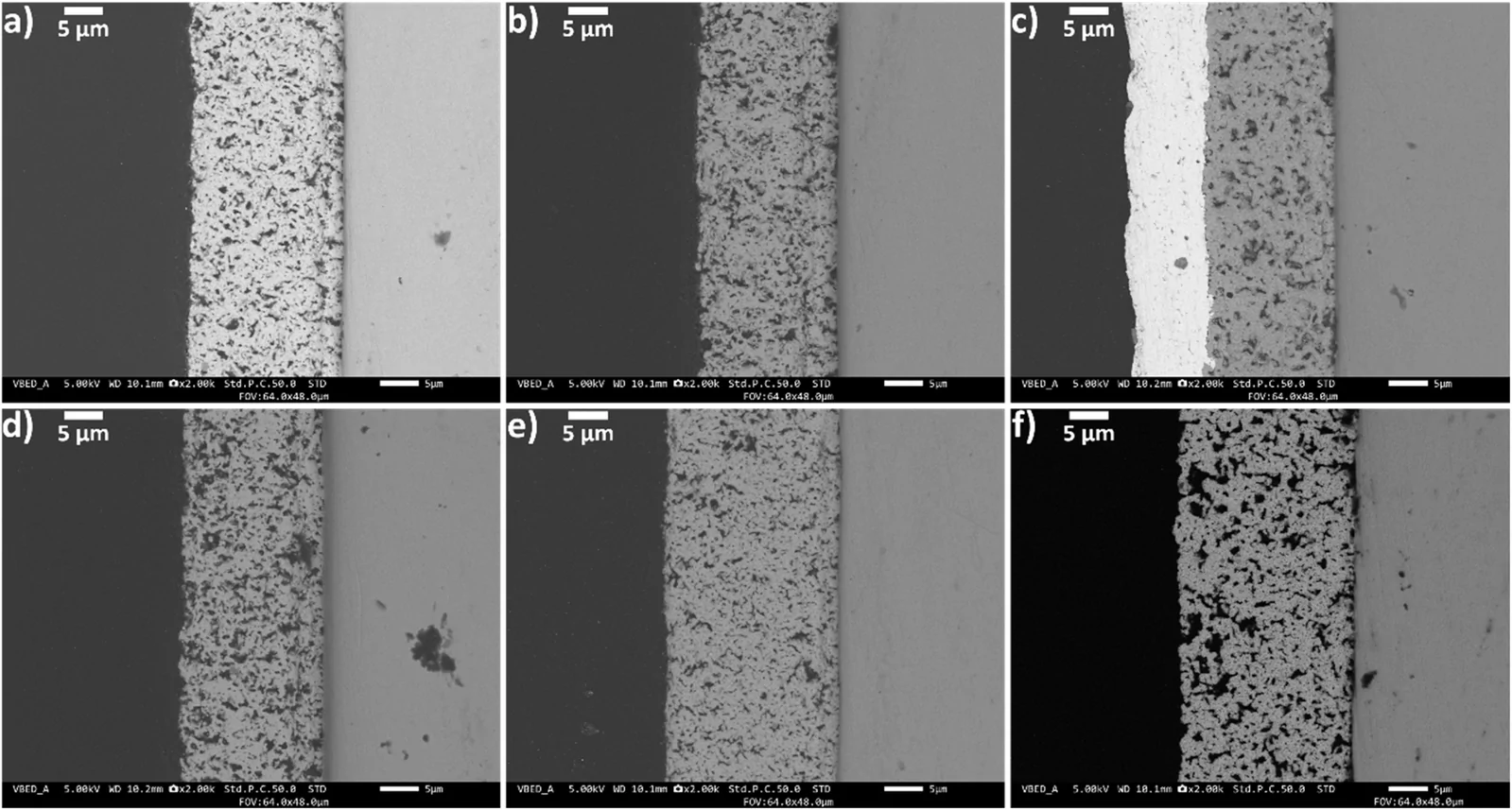
BSE micrographs of polished half-cell cross-sections for (a) LMO_A−_, (b) LMN_A−_, (c) LMF_A−_, (d) LMC_A−_, (e) LMT_A−_ and (f) LSM.

### Evaluation of solid oxide electrolysis cells containing robust electrodes for pure CO_2_ electrolysis in Martian environments

Given the low ‘effective’ electrical conductivity exhibited by the La_0.90_Mn_0.95_Ti_0.05_O_3_ sample in forming gas, these materials were investigated as current collectors for the fuel electrode of SOECs for carbon dioxide electrolysis, due to the less reducing gas composition. Although CO_2_ is only a weakly reducing gas (p(O_2_) ∼10^−6^ at 850 °C),^65^ the continuous generation of CO means the equilibrium gas composition becomes more reducing during operation.

Three SOECs were manufactured and tested in order to elucidate the role of different components, through observations of changes in performance between cells. All SOECs employed a 140 µm 5ScSZ electrolyte. SOECs 1 and 2 contain the same air electrode (LSC/CGO), whose LSC layer was left unsintered, and LSCT_A−_ fuel electrodes with CGO and Rh impregnates (25 µg cm^−2^ and 41 µg cm^−2^ of Rh for each cell, respectively). In addition, SOEC 1 contained a Ni paste current collector, whilst SOEC 2 contained an unsintered layer of LMT_A−_ as an alternative current collector. SOEC 3 contained a nominally identical fuel electrode to SOEC 2, though the air electrode was replaced with a fully sintered LNF/CGO microstructure impregnated with Pr_6_O_11_ nanocatalysts (540 µg cm^−2^).

Fig. 11a–c show *VI* curves for SOECs 1, 2 and 3, respectively, collected between 750 °C and 850 °C at 25 °C intervals, whilst Fig. 11d–f show the corresponding AC impedance spectra collected in the same temperature range at −1.5 V. As expected, there is a significant increase in performance at higher temperatures for all SOECs, as shown by the higher maximum current passage in the *VI* curves and the significant reduction in area specific resistance (ASR) in the AC impedance spectra. This occurs as a result of the thermal activation of: (i) the ionic conductivity of the electrolyte, reducing the series resistance (*R*_s_), and (ii) polarisation processes pertaining to the electrodes (*R*_p_). The *VI* curves demonstrate that a higher maximum current density is achieved with the Ni current collector at all temperatures. It is desirable to operate SOECs at thermoneutral voltage, at which potential the joule heating of the cell matches the thermal requirement for the conversion of CO_2_ to CO. Therefore, the heat generated by applying −1.47 V is equal to the heat energy consumed during the reaction, minimising the need for external heating and cooling and allowing the simplification of systems.^66^ At −1.5 V, close to the thermoneutral voltage, and 850 °C, the current densities achieved were −1.23 A cm^−2^, −0.79 A cm^−2^ and −0.87 A cm^−2^ (according to the upscan of the *VI* curves), whilst the ASR values were 0.45 Ω cm^2^, 0.77 Ω cm^2^ and ∼0.70 Ω cm^2^, respectively. Although the initial performances of SOECs 2 and 3 are quite similar due to the use of the same LMT current collection layer, substitution with a Ni layer significantly increases the performance of SOEC 1, comparatively.

**Fig. 11:**
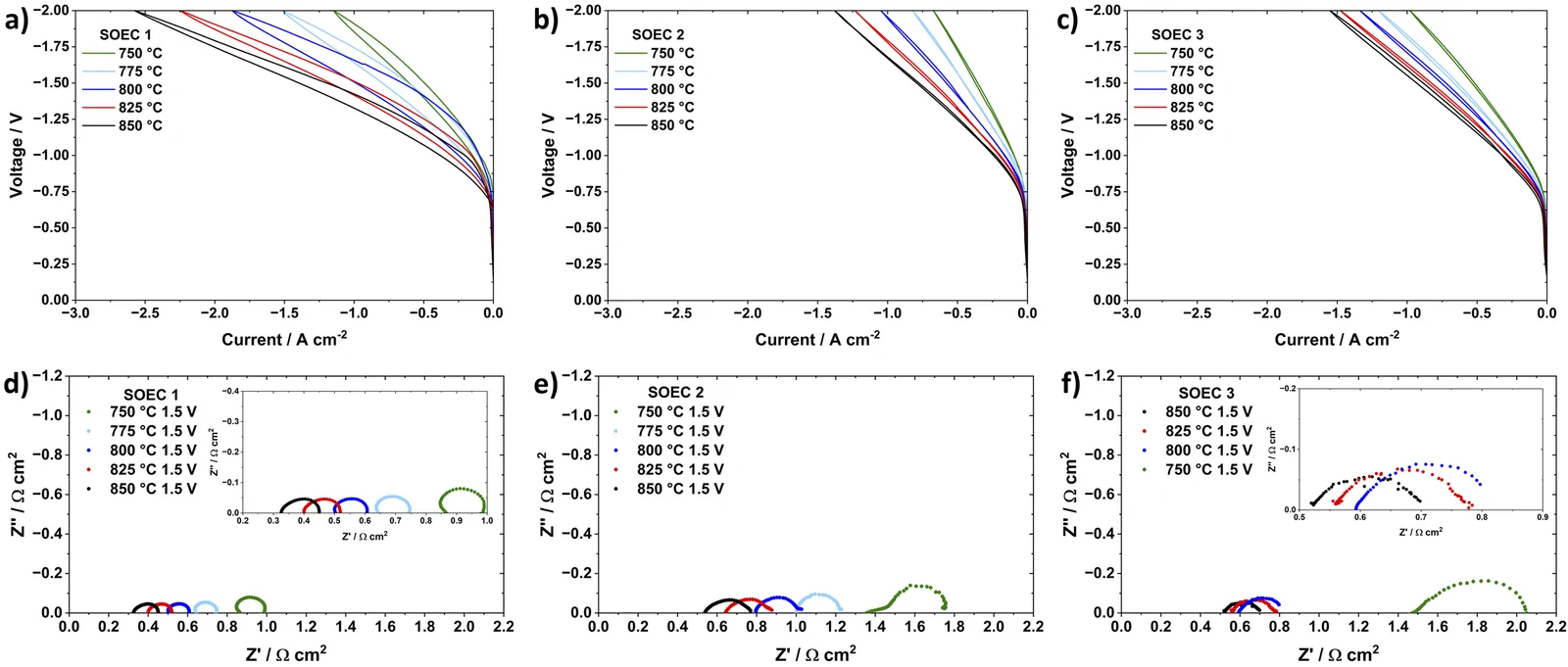
*VI* curves (a–c) and AC impedance spectra at −1.5 V (d–f) collected between 750 °C and 850 °C for SOEC 1 (Ni current collector), SOEC 2 (LMT current collector) and SOEC 3 (LMT current collector, with LNF-based air electrode), respectively.

In this work, equivalent circuit fitting (ECF) of AC impedance spectra was not performed, firstly, due to extensive previous fittings carried out on analogous SOECs containing LSM-10Sc1CeSZ/LSM air electrodes and, secondly, due to difficulties encountered with high-frequency inductive effects and low-frequency artefacts for SOEC 1. This arose due to a wiring issue within the test setup that was corrected for the following tests and caused masking of some processes within these frequency realms in comparison to SOEC 2, for example. In the authors' previous work, SOECs comprising a 100 µm 6ScSZ electrolyte, the LSCT_A−_ fuel electrodes with CGO and Rh impregnates and the aforementioned LSM-10Sc1CeSZ/LSM air electrode were tested under nominally identically conditions (850 °C, −1.5 V in pure CO_2_). Four polarisation processes were identified in the AC impedance response of these SOECs: (i) a charge transfer process in the fuel electrode (R_p1_, frequency maximum (*f*_max_) 400–500 Hz), (ii) a process relating to surface adsorption and diffusion of oxygen in the air electrode (R_p2_, *f*_max_ 60–180 Hz), (iii) a fuel electrode process possibly relating to surface adsorption and diffusion of CO_2_ in the fuel electrode (R_p3_, *f*_max_ 10–40 Hz) and (iv) a temperature independent gas diffusion process (R_p4_, *f*_max_ ∼6 Hz).

In order to determine whether SOEC 2 shares any of these impedance responses, distribution of relaxation times (DRT) analysis of the spectrum collected at 850 °C and −1.5 V (with 200 mL min^−1^ flow rates for both air and CO_2_) was performed (Fig. 12a). This is presented alongside Bode format AC impedance spectra collected under differing air and fuel flow rates (Fig. 12b and c, respectively) to help elucidate which processes belong to certain cell components. The DRT analysis indicates that four peaks (processes) are isolable; the processes with *f*_max_ = 30 Hz and 5 Hz align well with the previously noted R_p3_ (surface adsorption and diffusion of CO_2_) and R_p4_ (gas diffusion impedance) processes. In addition, the R_p1_ (fuel electrode charge transfer) process has shifted to a slightly lower *f*_max_ (∼200 Hz). This process does not relate to the aforementioned R_p2_ air electrode related process, due to the replacement of the performance limiting LSM-10Sc1CeSZ/LSM air electrode with LSC/CGO, whose impedance response is extremely small (see Fig. 4a). An additional process (*f*_max_ ∼0.7 Hz) can be seen in the low-frequency region of the DRT, which may relate to a combination of gas conversion impedance and chemical capacitance behaviour of the MIEC materials present in this SOEC (*i.e.*, CGO in both electrodes and LSC).

**Fig. 12:**
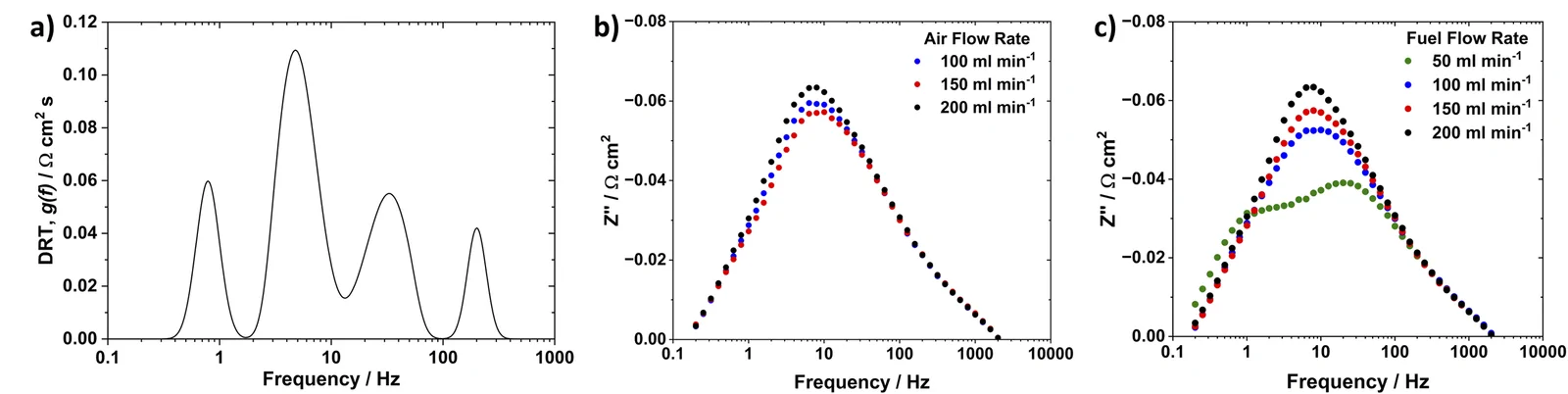
AC impedance spectra for SOEC 2 collected at 850 °C and −1.5 V after DRT analysis (a) and Bode format spectra collected as a function of (b) air flow rate and (c) fuel flow rate.

This response is particularly prominent in SOFCs containing the same fuel electrode material. Fig. 12b indicates that there is not a significant change in impedance response when reducing the air flow rate from 200 mL min^−1^ to 100 mL min^−1^, whereas reduction of the fuel flow rate from 200 mL min^−1^ to 50 mL min^−1^ (Fig. 12c) sequentially reduces polarisation resistance of the surface adsorption and diffusion process for CO_2_ in the fuel electrode, adding weight to this assignment of process. The resultant increase in fuel utilisation increases the proportion of CO in the fuel mixture locally within the fuel electrode, which may help to reduce this electrode and improve its electrocatalytic activity. The consequence of this increase in reducing gas content of the fuel stream for the stability of the LMT_A−_ fuel-side current collector has not yet been investigated; however, work by Ouweltjes *et al.* suggests that the lanthanum manganite materials could be stable in up to 10% CO/90% CO_2_ fuel mixtures.^22^

Finally, durability testing of these SOECs was performed at 850 °C and −1.47 V (thermoneutral voltage for CO_2_ electrolysis) (Fig. 13a). Although SOEC 1, with the Ni current collector, shows the highest initial current density, its performance degrades more rapidly than that of SOEC 2, which instead utilises a LMT_A−_ current collector on the fuel electrode. Interestingly, in both data traces, after a stop–start cycle of potentiostatic operation (at 92 h for SOEC 1 and 211 h for SOEC 2), the operating current density recovers almost to the value recorded at the start of the test period. Replacement of the LSC/CGO air electrode for a variant comprising LNF/CGO with impregnated Pr_6_O_11_ particles (whilst continuing to use the LMT_A−_ fuel electrode current collector) results in an improvement of current density and stability for SOEC 3. However, although degradation is minimised, its underlying behaviour is similar to both SOECs 1 and 2. Furthermore, the AC impedance spectra (Fig. 13b) collected before and after durability testing indicate that SOEC 1 shows an increase in *R*_s_ and ASR, most likely pertaining to oxidation of the Ni layer and reduction in ‘effective’ conductivity of this current collector in the absence of a reducing gas in the fuel stream. In comparison, both SOECs with LMT_A−_ current collectors show greater stability and, in fact, reductions in *R*_s_ and ASR after the test period. It is common to see activation in novel perovskite titanate fuel electrodes under high applied electrolysis potentials over time;^67,68^ however, this does not reconcile with the degradation behaviour observed in Fig. 13a at first consideration.

**Fig. 13:**
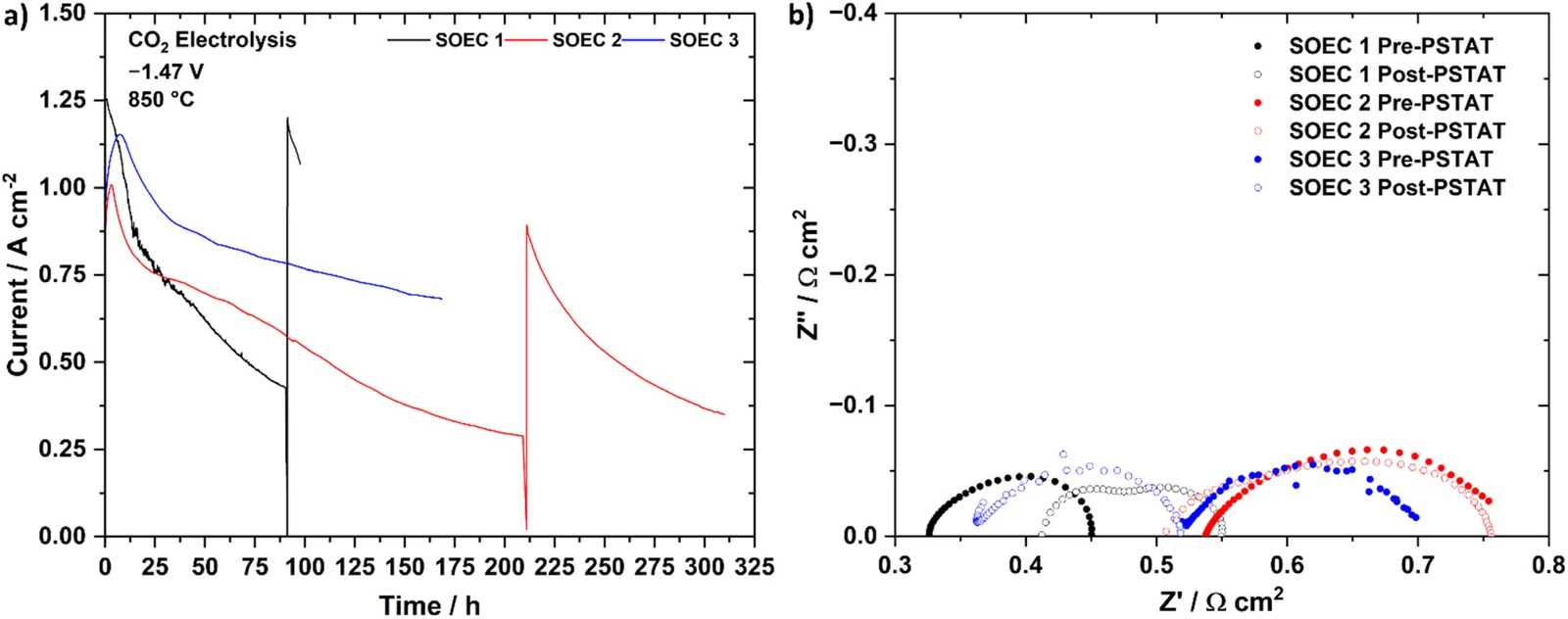
(a) Potentiostatic data for SOECs 1, 2 and 3 at −1.47 V for up to 315 h and (b) AC impedance spectra collected at −1.5 V and before and after this testing period for each SOEC, at 850 °C using a CO_2_ fuel gas.

As the AC impedance spectra were collected a short time after conclusion of the test period, during which the voltage relaxes and the current density appears to recover, the impedance response also reflects these comparatively relaxed conditions. Although the spectra in Fig. 13b show that there are some changes in the low-frequency region of *R*_p_, the recovery of performance is very encouraging for the durability of these SOECs. Further investigation is required to understand the origin of this ‘current decay’ during potentiostatic operation; however, it is tentatively suggested that the kinetics of CO_2_ adsorption and diffusion may be contributing to this,^69^ particularly as these processes have been identified as the rate-limiting steps in AC impedance spectroscopic studies on SOCs containing (La_0.75_Sr_0.25_)_0.97_Cr_0.5_Mn_0.5_O_3−*δ*_/CGO composite fuel electrodes.^70^

## Conclusions

This research demonstrated that high-performance, next-generation air electrode materials comprising LSM/CGO and LNF/CGO microstructures, impregnated with Pr_6_O_11_, offer superior performance and stability under harsh solid-oxide electrochemical oxygen pumping conditions than traditional material sets, such as composite LSM-stabilised zirconia and LSM-CGO, as well as LSC/CGO air electrodes. The improved robustness of these versatile devices provides clear benefits for the performance and durability of systems undertaking oxygen separation in terrestrial environments, *e.g.*, use of oxygen in medical applications or use of the nitrogen separated from air for the Haber–Bosch process, as well as in extra-terrestrial localities, where life support and propellant production processes are not only critical, but must be reliable and robust. In addition, the development of La_0.90_Mn_0.95_X_0.05_O_3_ materials, doped with transition metals on the B-site of the perovskite structure, indicated that La_0.90_Mn_0.95_Ti_0.05_O_3_ offered the greatest stability in ‘effective’ electrical conductivity under reducing conditions (p(O_2_) = 10^−18^–10^−19^ atm) and could be advantageous to use as a fuel electrode current collection layer for solid oxide electrolysis cells fed with pure CO_2_ (without the use of a reducing, safe gas). Implementation of this optimised fuel-side current collection layer, alongside the high-performance Pr_6_O_11_-impregnated LNF/CGO air electrode investigated through solid-oxide electrochemical oxygen pump testing, significantly improved the durability of SOECs operating with a pure CO_2_ fuel gas at 850 °C and thermoneutral voltage (−1.47 V) for almost 200 h, which is key for improving the resilience, robustness and complexity of electrolysis systems operating in both terrestrial and extra-terrestrial locations. Post-mortem evaluation of tested SOECs is currently underway to identify causes of degradation; however, further research is required in order to understand the gradual reduction in current density under the aforementioned conditions, which may relate to diffusion and kinetic limitations of CO_2_ adsorption and diffusion at the surface of the fuel electrode, and the consequent ‘regenerative’ behaviour of these SOECs following brief pauses in potentiostatic operation.

## Conflicts of interest

There are no conflicts of interest to declare.

## Data Availability

The data underpinning this work are available from the corresponding author upon reasonable request.

## References

[cit1] International Energy Agency , Global Hydrogen Review 2025, 2025

[cit2] Green R. D., Elangovan S. E., Chen F. (2022). J. Electrochem. Soc..

[cit3] Jackson G. S., Elangovan S., Hintze P. E. (2020). Electrochem. Soc. Interface.

[cit4] Singh M., Zappa D., Comini E. (2021). Int. J. Hydrogen Energy.

[cit5] Hauch A., Küngas R., Blennow P., Hansen A. B., Hansen J. B., Mathiesen B. V., Mogensen M. B. (2020). Science.

[cit6] Hauch A., Jensen S. H., Ramousse S., Mogensen M. (2006). J. Electrochem. Soc..

[cit7] Liu H., Yu M., Tong X., Wang Q., Chen M. (2024). Chem. Rev..

[cit8] Detz R. J., Ferchaud C. J., Kalkman A. J., Kemper J., Sánchez-Martínez C., Saric M., Shinde M. V. (2023). Sustainable Energy Fuels.

[cit9] Wolf S. E., Winterhalder F. E., Vibhu V., de Haart L. G. J., Guillon O., Eichel R. A., Menzler N. H. (2023). J. Mater. Chem. A.

[cit10] Takada K. , Ridley A., Van Keuren S., Baker P. S. and McDougle S. H., in Proc. 50th International Conference on Environmental Systems, ICES-2021-264

[cit11] Rapp D., Inglezakis V. J. (2024). Appl. Sci..

[cit12] Starr S. O., Muscatello A. C. (2020). Planet. Space Sci..

[cit13] Sapountzi F. M., Gracia J. M., (Kees-Jan) Weststrate C. J., Fredriksson H. O. A., (Hans) Niemantsverdriet J. W. (2017). Prog. Energy Combust. Sci..

[cit14] Igwe C. I., Achebe C. H., Chinweze A. E. (2022). Global Sci. J..

[cit15] Wang X. , Hu L., Yang M., Han Z. and Yang Z., in Green Hydrogen Production by Water Electrolysis, ed. J. Hou and M. Yang, CRC Press, Boca Raton, USA, 2024, pp. 320–344

[cit16] Jiang S. P. (2016). Asia-Pac. J. Chem. Eng..

[cit17] Momma A., Kato T., Kaga Y. (1997). J. Ceram. Soc. Jpn..

[cit18] Ormerod R. M. (2003). Chem. Soc. Rev..

[cit19] Mahata A., Datta P., Basu R. N. (2017). Ceram. Int..

[cit20] Wood T. and Ivey D. G., in Solid Oxide Fuel Cell Lifetime and Reliability: Critical Challenges in Fuel Cells, ed. N. P. Brandon, E. Ruiz-Trejo and P. Boldrin, Elsevier, London, UK, 2017, pp. 51–77

[cit21] Faes A., Hessler-Wyser A., Zryd A., Van Herle J. (2012). Membranes.

[cit22] Ouweltjes J. P., Van Tuel M., Sillessen M., Rietveld G. (2009). Fuel Cells.

[cit23] Sun C., Stimming U. (2007). J. Power Sources.

[cit24] Price R., Cassidy M., Grolig J. G., Longo G., Weissen U., Mai A., Irvine J. T. S. (2021). Adv. Energy Mater..

[cit25] Wang P. , Wang J., Fu L., Wu Y. and van Ree T., in Metal Oxides in Energy Technologies, ed. Y. Wu, Elsevier, Amsterdam, 2018, pp. 17–47

[cit26] Moçoteguy P., Brisse A. (2013). Int. J. Hydrogen Energy.

[cit27] Battaia O. , Oloruntoba R. and Guillaume R., in Proceedings of the Conference on Production Systems and Logistics, Publish-Ing in cooperation with TIB - Leibniz Information Centre for Science and Technology University Library, 2024, pp. 838–852

[cit28] Aljaberi A. D., Irvine J. T. S. (2013). J. Mater. Chem. A.

[cit29] Verbraeken M. C., Ramos T., Agersted K., Ma Q., Savaniu C. D., Sudireddy B. R., Irvine J. T. S., Holtappels P., Tietz F. (2015). RSC Adv..

[cit30] Gavin A. L., Watson G. W. (2018). Phys. Chem. Chem. Phys..

[cit31] Price R., Fuente Cuesta A., Bausinger H., Longo G., Naden A. B., Grolig J. G., Mai A., Irvine J. T. S. (2024). J. Electrochem. Soc..

[cit32] Siebenhofer M., Viernstein A., Morgenbesser M., Fleig J., Kubicek M. (2021). Mater. Adv..

[cit33] Price R., Bausinger H., Longo G., Weissen U., Cassidy M., Grolig J. G., Mai A., Irvine J. T. S. (2023). Fuel Cells.

[cit34] Hughes G. A., Railsback J. G., Yakal-Kremski K. J., Butts D. M., Barnett S. A. (2015). Faraday Discuss..

[cit35] Wang M., Nowicki K. M., Irvine J. T. S. (2022). J. Electrochem. Soc..

[cit36] Hafen T., Rane T., Larsen D., Pike J., Hartvigsen J., Elwell J., Coyle C., Marina O. A., Elangovan S. (2023). ECS Trans..

[cit37] Hendriksen P. V., Sun X., Mondi F., Monich P. R., Pirou S., Frandsen H. L. (2023). ECS Trans..

[cit38] Heaney M. B. , in Electrical Measurement, Signal Processing, and Displays, ed. J. G. Webster, CRC Press, Boca Raton, 2003, pp 1–14

[cit39] Tietz F., Sebold D., Brisse A., Schefold J. (2013). J. Power Sources.

[cit40] Chen K., Jiang S. P. (2016). J. Electrochem. Soc..

[cit41] Fan Y., Chen Y., Abernathy H., Pineault R., Addis R., Song X., Hackett G., Kalapos T. (2023). J. Power Sources.

[cit42] Sun X., Chen M., Liu Y.-L., Hjalmarsson P., Ebbesen S. D., Jensen S. H., Mogensen M. B., Hendriksen P. V. (2013). J. Electrochem. Soc..

[cit43] Graves C., Ebbesen S. D., Mogensen M. (2011). Solid State Ionics.

[cit44] Huber A. K., Falk M., Rohnke M., Luerssen B., Amati M., Gregoratti L., Hesse D., Janek J. (2012). J. Catal..

[cit45] Mai A., Haanappel V. A. C., Uhlenbruck S., Tietz F., Stöver D. (2005). Solid State Ionics.

[cit46] Tu H. Y., Takeda Y., Imanishi N., Yamamoto O. (1999). Solid State Ionics.

[cit47] Seo H., -eun Won J., Lee W., Choi H., Park S. Y., Shin Y., Lee I., Lim T. J., Kim K., Hong J., Chang H. J., Yoon K. J. (2025). Adv. Sci..

[cit48] Adijanto L., Küngas R., Bidrawn F., Gorte R. J., Vohs J. M. (2011). J. Power Sources.

[cit49] Shah M., Barnett S. A. (2008). Solid State Ionics.

[cit50] Jiang S. P. (2006). Mater. Sci. Eng., A.

[cit51] Nicollet C., Flura A., Vibhu V., Rougier A., Bassat J.-M., Grenier J.-C. (2016). Int. J. Hydrogen Energy.

[cit52] Mauvy F., Grenier J. C., Vulliet J., Bassat J. M., Rougier A. (2022). Fuel Cells.

[cit53] Navarrete L., Solís C., Serra J. M. (2015). J. Mater. Chem. A.

[cit54] Orera A., Betato A., Silva-Treviño J., Larrea Á., Laguna-Bercero M. (2022). J. Mater. Chem. A.

[cit55] Shen Q., Tong Y., Chen C., Zhan Z. (2023). Sep. Purif. Technol..

[cit56] Konysheva E., Irvine J. T. S. (2009). J. Power Sources.

[cit57] Liu S. (2015). Phys. B.

[cit58] Yang J., Ma Y. Q., Zhang R. L., Zhao B. C., Ang R., Song W. H., Sun Y. P. (2005). Solid State Commun..

[cit59] Rahaman M. N. , in Kirk-Othmer Encyclopedia of Chemical Technology, John Wiley & Sons, Hoboken, USA, 2014, pp. 1–98

[cit60] Mistler R. and Twiname E., Tape Casting: Theory and Practice, The American Ceramic Society, Westerville, USA, 2000

[cit61] Price R., Cassidy M., Schuler J. A., Mai A., Irvine J. T. S. (2017). ECS Trans..

[cit62] Carter C. B. and Norton M. G., Ceramic Materials, Springer, New York, USA, 2014

[cit63] Kong J. , Sun K., Zhou D., Qiao J. and Li J., in International Forum on Strategic Technology, IEEE, 2006, pp.186–189

[cit64] Vibhu V., Vinke I. C., Eichel R. A., de Haart L. G. J. (2023). Energies.

[cit65] Oleksak R. P. and Rouillard F., in Comprehensive Nuclear Materials, Elsevier, 2nd edn, 2020, pp. 422–451

[cit66] Hou X., Jiang Y., Wei K., Jiang C., Jen T. C., Yao Y., Liu X., Ma J., Irvine J. T. S. (2024). Chem. Rev..

[cit67] Myung J., Neagu D., Miller D. N., Irvine J. T. S. (2016). Nature.

[cit68] Chanthanumataporn M., Hui J., Yue X., Kakinuma K., Irvine J. T. S., Hanamura K. (2019). Electrochim. Acta.

[cit69] Green R. D., Liu C. C., Adler S. B. (2008). Solid State Ionics.

[cit70] Yue X., Irvine J. T. S. (2012). Electrochem. Solid-State Lett..

